# Noninvasive detection of any-stage cancer using free glycosaminoglycans

**DOI:** 10.1073/pnas.2115328119

**Published:** 2022-12-05

**Authors:** Sinisa Bratulic, Angelo Limeta, Saeed Dabestani, Helgi Birgisson, Gunilla Enblad, Karin Stålberg, Göran Hesselager, Michael Häggman, Martin Höglund, Oscar E. Simonson, Peter Stålberg, Henrik Lindman, Anna Bång-Rudenstam, Matias Ekstrand, Gunjan Kumar, Ilaria Cavarretta, Massimo Alfano, Francesco Pellegrino, Thomas Mandel-Clausen, Ali Salanti, Francesca Maccari, Fabio Galeotti, Nicola Volpi, Mads Daugaard, Mattias Belting, Sven Lundstam, Ulrika Stierner, Jan Nyman, Bengt Bergman, Per-Henrik Edqvist, Max Levin, Andrea Salonia, Henrik Kjölhede, Eric Jonasch, Jens Nielsen, Francesco Gatto

**Affiliations:** ^a^Department of Biology and Biological Engineering, Chalmers University of Technology, Gothenburg 412 96, Sweden; ^b^Department of Translational Medicine, Division of Urological Cancers, Lund University, 20502, Lund 205 02, Sweden; ^c^Department of Surgical Sciences, Uppsala University Hospital, Uppsala 751 85, Sweden; ^d^Department of Immunology, Genetics and Pathology, Uppsala University, Uppsala 751 85, Sweden; ^e^Department of Women's and Children’s, Health Uppsala University, Uppsala 751 85, Sweden; ^f^Department of Neurosurgery, Uppsala University Hospital, Uppsala 751 85, Sweden; ^g^Department of Urology, Uppsala University Hospital, Uppsala 751 85, Sweden; ^h^Institution of Medical Sciences, Uppsala University Hospital, Uppsala 751 85, Sweden; ^i^Department of Cardiothoracic Surgery and Anesthesiology, Uppsala University Hospital, Uppsala 751 85, Sweden; ^j^Department of Clinical Sciences Lund, Section of Oncology and Pathology, Lund University, Lund 221 85, Sweden; ^k^Department of Molecular and Clinical Medicine, Institute of Medicine Wallenberg Laboratory, Sahlgrenska Academy, University of Gothenburg, Gothenburg 413 45, Sweden; ^l^Vancouver Prostate Centre, Vancouver, BC V6H 3Z6, Canada; ^m^Department of Urologic Sciences, University of British Columbia, Vancouver, BC V5Z 1M9, Canada; ^n^Division of Experimental Oncology/Unit of Urology, Urological Research Institute, Istituto di Ricovero e Cura a Carattere Scientifico, Ospedale San Raffaele, Milan 20132, Italy; ^o^Department of Cellular and Molecular Medicine, University of California San Diego, La Jolla, CA 92093; ^p^Centre for Medical Parasitology at Department for Immunology and Microbiology, Faculty of Health and Medical Sciences, University of Copenhagen, Copenhagen 2200, Denmark; ^q^Department of Infectious Disease Copenhagen, University Hospital, Copenhagen 2300, Denmark; ^r^Department of Life Sciences, University of Modena and Reggio Emilia, Modena 411 25, Italy; ^s^Department of Urology, Institute of Clinical Sciences, Sahlgrenska Academy, University of Gothenburg, Gothenburg 413 45, Sweden; ^t^Department of Oncology, Institute of Clinical Sciences, Sahlgrenska Academy, University of Gothenburg, Gothenburg 413 45, Sweden; ^u^Department of Respiratory Medicine, Sahlgrenska Academy, University of Gothenburg, Gothenburg 413 45, Sweden; ^v^Università Vita-Salute San Raffaele, Milan 201 32, Italy; ^w^Department of Urology, Sahlgrenska University Hospital, Gothenburg 413 45, Sweden; ^x^The University of Texas MD Anderson Cancer Center, Houston, TX 77030; ^y^BioInnovation Institute, Copenhagen 2200, Denmark; ^z^Department of Oncology-Pathology, Karolinska Institute, Stockholm 171 64, Sweden; ^aa^Department of Urology, Kristianstad Central Hospital, Region Skåne, Kristianstad 291 33, Sweden

**Keywords:** cancer biomarkers, liquid biopsy, multi-cancer early detection, prognosis, metabolomics

## Abstract

Cancer mortality is exacerbated by late-stage diagnosis. Liquid biopsies based on genomic biomarkers can noninvasively diagnose cancers. However, validation studies have reported ~10% sensitivity to detect stage I cancer in a screening population and specific types, such as brain or genitourinary tumors, remain undetectable. We investigated urine and plasma free glycosaminoglycan profiles (GAGomes) as tumor metabolism biomarkers for multi-cancer early detection (MCED) of 14 cancer types using 2,064 samples from 1,260 cancer or healthy subjects. We observed widespread cancer-specific changes in biofluidic GAGomes recapitulated in an in vivo cancer progression model. We developed three machine learning models based on urine (*N_urine_* = 220 cancer vs. 360 healthy) and plasma (*N_plasma_* = 517 vs. 425) GAGomes that can detect any cancer with an area under the receiver operating characteristic curve of 0.83–0.93 with up to 62% sensitivity to stage I disease at 95% specificity. Undetected patients had a 39 to 50% lower risk of death. GAGomes predicted the putative cancer location with 89% accuracy. In a validation study on a screening-like population requiring ≥ 99% specificity, combined GAGomes predicted any cancer type with poor prognosis within 18 months with 43% sensitivity (21% in stage I; *N* = 121 and 49 cases). Overall, GAGomes appeared to be powerful MCED metabolic biomarkers, potentially doubling the number of stage I cancers detectable using genomic biomarkers.

## Significance

Multi-cancer early detection (MCED) is an emerging paradigm to curb cancer mortality by shifting stage at diagnosis through a single test capturing all cancer types when still confined to their tissue of origin (stage I). Liquid biopsies based on genomic biomarkers could make MCED realistic, but limitations include ~10% stage I sensitivity in validation studies; the inability to detect specific types like gliomas; complex assays; and potential overdiagnosis. Here, we demonstrate the potential of free glycosaminoglycan profiles (GAGomes) as metabolic biomarkers for MCED across 14 cancer types. In a validation study, plasma and urine GAGomes doubled the stage I sensitivity reported by state-of-the-art genomics biomarkers and detected poor prognosis cancers. As such, this simple assay could accelerate MCED implementation.

Early cancer detection is generally considered an effective strategy for reducing patient mortality. Screening programs for breast, prostate, lung, colorectal, and cervical cancer have significantly reduced mortality rates (1). However, there are currently no approved biomarkers for the early detection of most cancer types (2).

In recent years, significant advances have been made toward developing liquid biopsy platforms for universal cancer screening, or multi-cancer early detection (MCED), using noninvasive biofluidic biomarkers (3, 4). These platforms typically rely on sequencing and detecting cancer-derived fractions of circulating free DNA (cfDNA) (5). However, considerable challenges impede liquid biopsies. First, they mainly interrogate a specific layer of biological information about cancer, namely genomics. Second, some cancer types do not shed measurable cfDNA levels. A recent study (6, 7) classified 12 cancer types as “high-signal.” The remaining >30 cancer types account for 50% of global cases and are responsible for one-third of all cancer deaths. We noted that cancer types such as genitourinary and brain remained almost undetectable using cfDNA. Third, sensitivity to stage I cancer, small tumors that have not yet grown deep into nearby tissues, lymph nodes, or other body parts, remains far from ideal. Stage I sensitivity is critical for shifting to early, potentially curable, stage cancers, which is generally considered a prerequisite for reducing mortality through screening. Fourth, it remains unclear whether cancers detected by liquid biopsy screening have a poor prognosis. That is, whether they are potentially clinically significant instead of resulting in overdiagnosis and increasing screening’s harm/benefit ratio. Some of these challenges have been addressed by combining multiple information sources (e.g., DNA and proteins) to specifically detect distinct cancer types (8) by focusing on DNA fragmentation patterns instead of gene driver variants (9), enriching tumor-specific DNA methylation patterns (6, 10, 11), or reducing signal interference from clonal hematopoiesis (12). Generally, this approach has improved stage I sensitivity, sometimes as high as 70% (9). However, the complex assays required for these advanced liquid biopsies have increased costs that may be prohibitive for nationwide screening of the general population. Importantly, only a few liquid biopsies have validated their stage I sensitivity in an external representative population for MCED. The most extensively validated liquid biopsy based on targeted methylated cfDNA reported 16.8% stage I sensitivity in an external population (7). However, this may be overestimated since the study included symptomatic cancer cases, which are not representative of the MCED population. The only study validating a liquid biopsy for MCED in a screening-like external population detected 5 of 49 stage I cancers, giving it a stage I sensitivity of 10.2% (13).

Instead of genomics and proteomics, we investigated cancer metabolism as an identifiable cancer hallmark that could fill the information gap of current liquid biopsy platforms (14, 15). A systems biology pan-cancer analysis of tumor metabolism identified cancer-specific reprogramming of glycosaminoglycan (GAG) biosynthesis (16, 17). GAGs are a class of polysaccharides with remarkable structural diversity, reflecting complex sulfation and epimerization patterns arising during their template-free biosynthesis (18). The biological functions of GAGs include modulation of the extracellular matrix, cell proliferation and metabolism, and immune supervision (19, 20). Initially, we observed that plasma and urine free GAG profiles (GAGomes) were significantly altered in renal cell carcinoma (RCC), a genitourinary tumor, at any stage from organ-confined to metastatic disease (17, 21–23). This finding prompted us to investigate free GAGomes across different cancer types and develop a standardized high-throughput ultra-high-performance liquid chromatography coupled with triple-quadrupole mass spectrometry (UHPLC-MS/MS) method to measure free GAGomes (24).

In this study, we explored whether plasma and urine free GAGomes deviated from baseline physiological levels in 14 cancer types and could serve as metabolic cancer biomarkers. Next, we validated using free GAGomes for MCED in an external population, generating the largest compendium of biofluidic free GAGomes to date with 2,064 samples from 1,260 cancer patients and healthy subjects.

## Study Design and Free GAGome Measurements

**Fig. 1.**
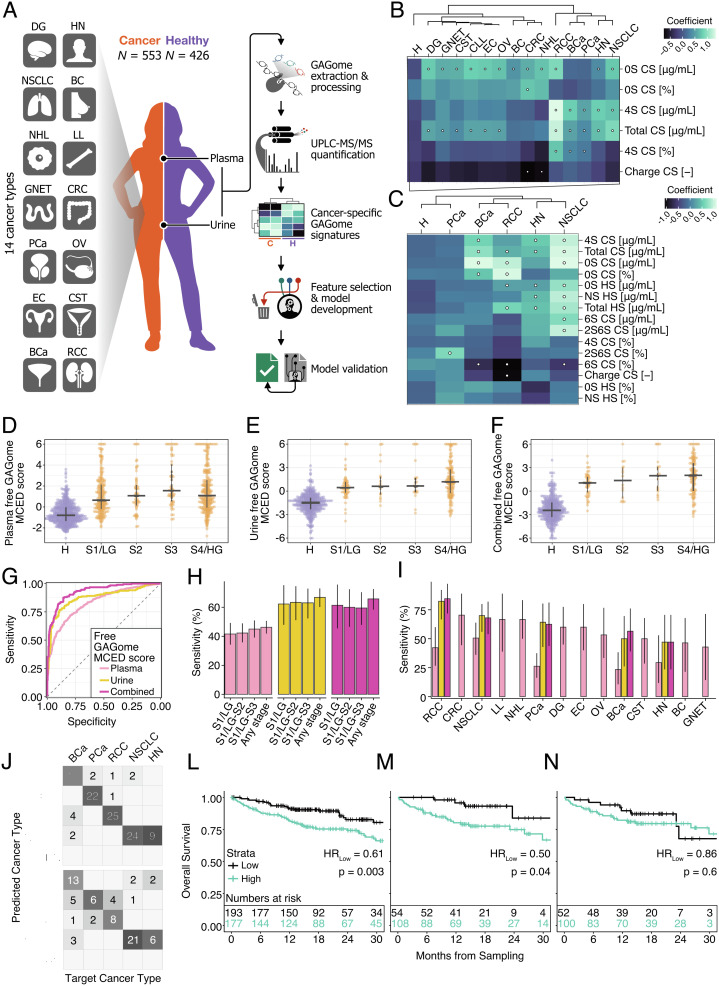
Development of plasma, urine, and combined free GAGome MCED scores. (*A*) Development study overview and summary of free GAGome analysis (*N*_tot_ = 979, 553 cancers vs. 426 healthy; *N*_plasma_ = 517 cancers vs. 425 healthy; *N*_urine_ = 220 cancers vs. 340 healthy; *N*_combined_ = 184 cancers vs. 339 healthy). (*B* and *C*) Estimated means of plasma and urine GAGome features conditional on the cancer type (median *N*_plasma_ per cancer type = 30, range: 14–83; median *N*_urine_ = 50 per cancer type, range: 17–56). Dots signify credible deviations from healthy subjects (i.e., from baseline physiological levels defined by ROPE criteria [see *Methods*]). The vertical axis denotes independent and dependent GAGome features (measured in μg mL^-1^ [except for charge, which is a.u.] or %w/w, respectively). (*D–F*) Free GAGome MCED scores across different stage/grade groups for plasma (*N_H_*= 425, *N_S1/LG_*= 178, *N_S2_*= 54, *N_S3_*= 57, and *N_S4/HG_*= 217), urine (*N_H_*= 340, *N_S1/LG_*= 53, *N_S2_*= 18, *N_S3_*= 21, and *N_S4/HG_*= 126), and for combined (*N_H_*= 339, *N_S1/LG_*= 44, *N_S2_*= 16, *N_S3_*= 19, and *N_S4/HG_*= 105). The crossbar denotes the median and 25th/75th quantiles. Cancers with unspecified stage/grade were omitted (*N* = 11 and 2 for plasma and urine, respectively). Scores were capped to the interval (−6,6); see *SI Appendix*, Table S5 for non-visualized data points). (*G*) ROC curves for plasma, urine, and combined scores in the discrimination of cancers vs. healthy (*N* as in panel *A*). (*H*) Sensitivity at 95% specificity for the plasma, urine, and combined scores across different stage/grade groups (*N* as in panels *D*–*F*). Colors as in *G*. Error bars denote the 95% CI boundaries. (*I*) Sensitivity at 95% specificity for the plasma, urine, and combined scores across different cancer types (*N* as in *SI Appendix*, Table S4). Error bars denote the 95% CI boundaries. Colors as in *G*. (*J* and *K*) Cancer-type prediction using a Bayesian Additive Regression Trees model in the training (*N* = 110, five cancer types) and test (*N* = 74, five cancer types) sets. The numbers in the boxes represent the number of samples classified as belonging to the predicted cancer type. (*L–N*) Kaplan–Meier curves for OS across all cancer patients stratified into groups of “low” (undetected) vs. “high” (detected) plasma (*N* = 370, 13 cancer types), urine (*N* = 162, four cancer types), and combined (*N* = 152, four cancer types) scores. For each score, patients with scores greater than the 95% specificity cutoff were assigned to the “high” (black) vs. “low” (cyan) group. Hazard ratios for patients belonging to the “low” score strata and log-rank test *P*-values are shown under each curve. The panels show the number at risk for each group. Key: H, healthy; S1/LG, stage I or low grade; S2, stage II; S3, stage III; S4/HG, stage IV or high grade; see Table 1 for cancer types.

We first conducted a case-control development study that included 979 subjects, 553 cancer patients representing 14 cancer types (Fig. 1*A*; *N* = 14–104, median per type = 28), and 426 healthy subjects, with similar demographic characteristics taken from multi-site international cohorts (Sweden and Italy; Table 1 and Dataset S1). Thirty-four percent of cancer patients were classified as stage I/low grade (6 to 66% across types, median per type = 41%; see *methods* for descriptions of staging and grading criteria; see *SI Appendix*, Table S1 for the number of samples across stages/grades). We measured the free GAGome in plasma from 96% of subjects (*N* = 942, 517 cancer and 425 healthy) and in urine from 57% of subjects (*N* = 560, 220 cancer and 340 healthy; all but one had a matched plasma GAGome) using standardized UHPLC-MS/MS kits in a single-blinded central laboratory (24). The free GAGome comprised the concentration of 17 disaccharide subunits of chondroitin sulfate (CS), heparan sulfate (HS), and hyaluronic acid (HA) (*SI Appendix*, Fig. S1 and Table S2) and calculated GAGome features (*SI Appendix*, Figs. S3 and S5), such as CS and HS charge, resulting in 39 total features per fluid. Of these 39 features, we found that six plasma and 17 urine GAGome features were detectable since their mean concentration was >0.1 µg mL^−1^.

**Table 1.** Population characteristics in the development (left) and validation (right) study

Development study (N = 979)			Validation study (N = 281)		
	Controls	Cases		Controls	Cases
	Healthy subjects	Cancer patients		No cancer in 18 mo	Cancer in 18 mo
N	426	553	N	110	171
Age	59 [22, 78]	67 [21, 91]	Age	60 [25, 84]	62 [29, 81]
Sex			Sex		
Female	246 (57.7%)	253 (45.8%)	Female	50 (45.5%)	87 (50.9%)
Male	180 (42.3%)	300 (54.2%)	Male	60 (54.5%)	84 (49.1%)
Sample availability			Sample availability		
Plasma only	86 (20.2%)	333 (60.2%)	Plasma and urine	110 (100%)	171 (100%)
Plasma and urine	339 (79.6%)	184 (33.3%)	Blood chemistry		
Urine only	1 (0.2%)	36 (6.5%)	CRP (mg/dL)	1.3 [0.3–14.9]	1.5 [0.2–19.3]
Tumor stage/grade			HDL-C (mg/dL)	1.4 [0.8–2.3]	1.4 [0.8–2.8]
Stage I/low grade*		187 (33.8 %)	Tumor stage^†^		
Stage II*		56 (10.1%)	Stage 0		13 (7.6%)
Stage III*		59 (10.7%)	Stage I		59 (34.5%)
Stage IV/high grade*		238 (43%)	Stage II		35 (20.5%)
Unspecified stage/grade		13 (2.4%)	Stage III		33 (19.3%)
Tumor histology			Stage IV		18 (10.5%)
Breast cancer (BC)		28 (5.1%)	Unspecified stage		13 (7.6%)
Bladder cancer (BCa)		47 (8.5%)	Tumor histology^†^		
Chronic lymphocytic leukemia (CLL)		18 (3.3%)	Breast cancer (BC)		45 (26.3%)
Cervical cancer (CST)		28 (5.1%)	Colorectal cancer (CRC)		40 (23.3%)
Colorectal cancer (CRC)		27 (4.9%)	Gynecological cancers (CST/EC/OV)		11 (6.4%)
Diffuse glioma (DG)		40 (7.2%)	Non-small cell lung cancer (NSCLC)		18 (10.5%)
Diffuse large B cell lymphoma (NHL)		30 (5.4%)	Prostate cancer (PCa)		42 (24.6%)
Endometrial carcinoma (EC)		30 (5.4%)	Urinary tract cancers (BCa/RCC)		15 (8.8%)
Head and neck cancer (HN)		17 (3.1%)	Time to diagnosis^†^		
Non-small cell lung cancer (NSCLC)		83 (15.0%)	< 3 mo		35 (20.5%)
Ovarian carcinoma (OV)		30 (5.4%)	3 to 18 mo		136 (79.5%)
Prostate cancer (PCa)		104 (18.8%)	Confirmation of cancer diagnosis^†^		
Renal cell carcinoma (RCC)		57 (10.3%)	Linkage to Dutch Cancer Registry		171 (100%)
Small intestinal neuroendocrine tumor (GNET)		14 (2.5%)			

Distributions are summarized as median and range. Key: CRP, high sensitivity C-reactive protein in heparin; HDL-C, high-density lipoprotein cholesterol in heparin.

*“Stage I/low grade” included cancers with TNM (8th edition), FIGO or Ann Arbor stage I, ENETS grade 1, Gleason grade <7, or non-grade IV glioma; “Stage IV/high-grade” included cancers with TNM (8th edition), FIGO or Ann Arbor stage IV, ENETS grade 2, Gleason grade ≥7, or grade IV glioma; “Stage II” and “Stage III” included cancers with the corresponding stage number in the TNM (8th edition) or FIGO or Ann Arbor systems. See SI methods for details on staging and grading criteria for grouping in the development study.

^†^Stage, time to diagnosis, and histology information were determined through linkage with the Dutch Cancer Registry. Note that self-reported cancer cases not confirmed by the Dutch Cancer Registry were excluded from the validation study (see *Methods*).

### Free GAGomes in Cancer and Healthy Subjects.

Next, we compared each detectable GAGome feature in each cancer type to their baseline physiological level in healthy subjects using a Bayesian mixed effect linear regression model with a skewed-normal response. The sensitivity analysis showed that model estimates were robust to the choice of prior width (*SI Appendix*, Fig. S2). We considered a GAGome feature meaningfully different from physiological levels for a given cancer type based on a region of practical equivalence (ROPE) centered on the level estimated in healthy subjects (Fig. 1 *B* and *C* and *SI Appendix*, Figs. S1 and S3, and Dataset S2). This analysis highlighted several GAGome features that deviated from physiological levels across multiple cancers (Fig. 1*B* and *SI Appendix*, Fig. S4). For example, we observed an almost universal increase in the urine and plasma concentration of non-sulfated CS (0S CS). We also identified several cancer-type-specific GAGome features, such as a lower plasma CS charge in colorectal cancer (CRC) and diffuse large B cell lymphoma (NHL), and elevated urine 2S6S CS in prostate cancer (PCa).

### Development of Free GAGome MCED Scores.

Having confirmed that all cancer types shared a universal GAGome feature signature that differed from baseline physiological levels, we explored using free GAGomes to robustly discriminate any cancer from healthy subjects. We used cross-validation projection predictive variable selection (25, 26) to develop three Bayesian logistic regression models to correlate any cancer vs. healthy subjects. The variable selection procedure converged on a minimal subset of informative GAGome features from plasma (*N_features_* = 3), urine (*N_features_* = 13), or both (*N_features_* = 14; *SI Appendix*, Fig. S6 and Dataset S3). Each model was internally validated by bootstrap resampling to control for overfitting (*SI Appendix*, Fig. S7). We defined each model’s log-predicted probability of any cancer as the plasma, urine, and combined free GAGome MCED score. The sensitivity analysis results suggested that the variable selection was robust to the prior choice. First, the variable selection procedure converged to a common set of top six features irrespective of the choice of prior width. Second, the alternative projected models resulted in scores highly correlated with the combined free GAGome MCED score (*SI Appendix*, Fig. S8).

For each of the three free GAGome MCED scores, we estimated metrics of discrimination (in terms of area under the receiver operating characteristic curve [ROC; AUC]) and clinical usefulness (sensitivity at 95% specificity). The scores distinguished any cancer from healthy subjects with an AUC = 0.83 (95% confidence interval [CI]: 0.80–0.86) for plasma (Fig. 1 *D*–*G* and *SI Appendix*, Fig. S9), AUC = 0.88 (95% CI: 0.85–0.91) for urine (Fig. 1 *E*–*G* and *SI Appendix*, Fig. S9), and AUC = 0.93 (95% CI: 0.90–0.95) for combined plasma and urine (Fig. 1 *F* and *G* and *SI Appendix*, Fig. S9). The sensitivity to any cancer for the plasma, urine, and combined free GAGome MCED score was 46.2% (95% CI: 41.9 to 50.6%), 66.8% (95% CI: 60.2 to 73.0%), and 65.8% (95% CI: 58.4 to 72.6%) at 95% specificity, respectively (Fig. 1*H*). At 99% specificity, the sensitivity was 25.7% (95% CI: 22.0 to 29.7%) for plasma, 25.0% (95% CI: 19.4 to 31.3%) for urine, and 35.3% (95% CI: 28.4 to 42.7%) for combined plasma and urine (*SI Appendix*, Table S3). In the stage I/low-grade disease subset, the sensitivity at 95% specificity was 41.6% (95% CI: 34.2 to 49.2%) for plasma, 62.3% (95% CI: 47.9 to 75.2%) for urine, and 61.4% (95% CI: 45.5 to 75.6%) for combined plasma and urine (*SI Appendix*, Table S3). All three scores showed a weak dependency between free GAGome alterations and tumor stage or grade, with a slight sensitivity increase in stages I–II and further in stages I–III (Fig. 1*H* and *SI Appendix*, Table S3). Overall, we observed a similar sensitivity of each score across individual cancer types (Fig. 1*I* and *SI Appendix*, Fig. S9 and Table S4). The top detected cancer types were NHL, CRC, and chronic lymphocytic leukemia (CLL) for the plasma score (range: 23.4% in bladder cancer [BCa] to 66.7% in NHL, CLL, and CRC) and RCC and non-small cell lung cancer (NSCLC) for the urine or combined scores (range: 47.1% in head and neck [HN] squamous cell carcinoma to 82.4 to 84.6% in RCC, for urine and combined, respectively). Altogether, these findings suggested that free GAGomes differed significantly from physiological levels across early- and late-stage cancers and could be used for MCED.

### Prediction of the Putative Cancer Location (PCL) using Free GAGomes.

Given the presence of distinctive free GAGomes across cancer types (Fig. 1 *B* and *C* and *SI Appendix*, Fig. S10), we explored whether these patterns could be used to identify the cancer type. We developed a multinomial Bayesian Additive Regression Trees model (27) using a training set (*N* = 110 across five cancer types) to predict the cancer type based on combined free GAGomes (Fig. 1*J*). Next, we validated the model’s accuracy in a test set (*N* = 74). The balanced classification accuracy was 74.3% (95% CI: 68.1 to 80.3%; Fig. 1*K*). We grouped tumors into two PCLs that would be actionable for location-specific diagnostic work-up: respiratory tract (NSCLC and HN) vs. genitourinary tumors (RCC, PCa, and BCa). The PCL prediction accuracy was 89.2% (95% CI: 82.2 to 96.4%).

### Correlation between Free GAGome MCED Scores and Overall Survival (OS).

To assess whether altered GAGome features associated with cancer were suggestive of aggressive tumor biology, we correlated each score with OS. From the date of sample collection, the median follow-up time was 17 mo in the plasma cohort (*N* = 370 across 13 cancer types, range: 14–47 per type; *N*_deaths_ = 82, range: 1–18 per type), 15 mo in the urine cohort (*N* = 162 across four cancer types, range: 17–50 per type; *N*_deaths_ = 33, range: 4–13 per type), and 15 mo in the combined cohort (*N* = 152 across four cancer types, range: 17–50 per type; *N*_deaths_ = 33, range: 4–13 per type). All three scores were independent OS predictors in a multivariable Cox regression (hazard ratio [HR] = 1.29, 95% CI: 1.06–1.56, and *P* = 0.0009 for plasma; HR = 1.79, 95% CI: 1.27–2.53, and *P* = 0.0009 for urine; HR = 1.91, 95% CI: 1.33–1.73, and *P* = 0.0004 for combined) after adjusting for cancer type, age, sex, and stage IV or high-grade disease. These findings associated free GAGome alterations with aggressive cancer phenotypes. Furthermore, they suggested that subjects with a score below the 95% specificity cutoff—i.e., undetected when using the free GAGome MCED scores—might have a better prognosis. To confirm this for each score, we dichotomized patients into “high” vs. “low” groups depending on whether their score was above or below the score-specific 95% specificity cutoff. For plasma and urine, Kaplan–Meier survival analyses suggested that “low” risk (undetected) patients had a 39 to 50% lower risk of death than “high” risk (detected) patients (HR = 0.61, 95% CI: 0.44–0.85, and *P* = 0.0031 in plasma [Fig. 1*L*]; HR = 0.50, 95% CI: 0.25–0.98, and *P* = 0.0441 in urine [Fig. 1*M*]). The survival difference between groups did not reach statistical significance for the combined score (HR = 0.86, 95% CI: 0.50–1.49, *P* = 0.5950; Fig. 1*N*). Importantly, we observed a lower risk of death in both the stage I-III/low-grade subset and the stage IV/high-grade subset, suggesting that the correlation between free GAGomes and prognosis was independent of the stage (*SI Appendix*, Fig. S11 *A*–*C*). In a sensitivity analysis, an alternative dichotomization with a “high” vs. “low” risk cutoff optimized using maximally selected rank statistics resulted in significant correlations between all three scores and OS across and within cancer types (Supplementary Note and *SI Appendix*, Figs. S12 and S13). Cumulatively, these survival analyses suggested that patients who would be undetected with the free GAGome MCED scores had a better prognosis and less aggressive cancer phenotype independent of tumor stage and grade.

### Validation of Free GAGome MCED Scores.

**Fig. 2.**
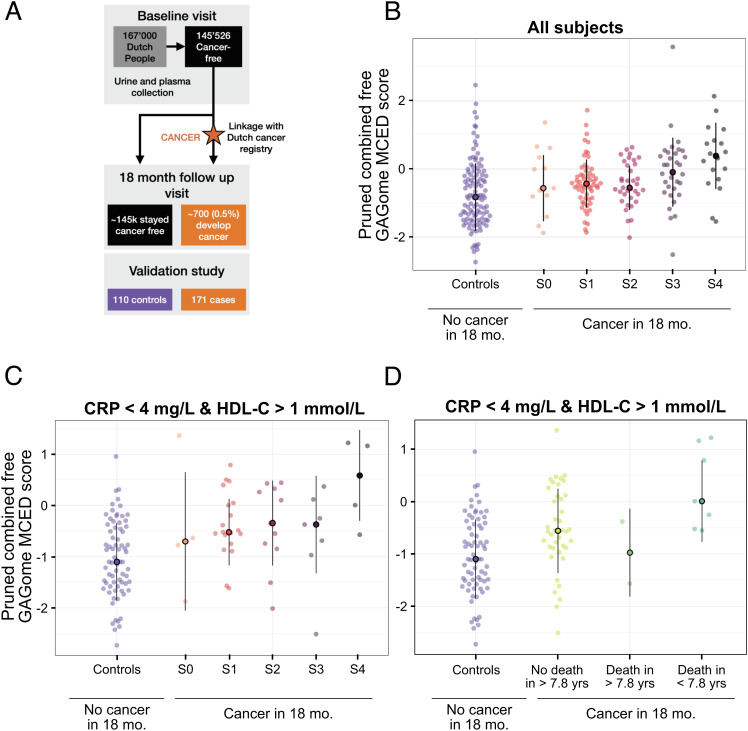
Validation of the pruned combined free GAGome MCED score. (*A*) Validation study subject flow (see also *SI Appendix*, Fig. S17). (*B*) Pruned combined free GAGome MCED scores across subjects with and without a cancer diagnosis 18 mo after the baseline visit (*N_tot_ =* 281, 110 controls vs. 158 cases; 13 cases with no stage information at diagnosis were omitted). Subjects that received a cancer diagnosis within 18 mo are grouped and colored according to the stage at diagnosis. The point and line range represent the median ± 1 standard deviation of the scores within each group. (*C* and *D*) Pruned combined free GAGome MCED scores in the subset of subjects with <4 mg/dL CRP and >1 mmol/L HDL-C (*N_tot_ =* 121, 72 controls vs. 49 cases; 4 cases with no stage information at diagnosis were omitted from panel *C*). Subjects that received a cancer diagnosis 18 mo after the baseline visit are grouped by the stage at diagnosis (C) and by time to death after diagnosis using the median overall survival (7.8 y) as cutoff for grouping (*D*). The point and line range represent the median ±1 standard deviation of the scores within each group. Key: S0, stage 0 (carcinoma in situ); S1, stage I; S2, stage II; S3, stage III; S4, stage IV.

We sought to confirm whether the free GAGome MCED scores could be used in a representative population for screening. We focused on the combined GAGomes as the best performing test and, before validation, we repeated the variable selection limited only to independently measured GAGome features as we reasoned that focusing on less as well as independently measured variables would increase test robustness and facilitate assay reproducibility. This step pruned the model size from 14 to 5 GAGome features (plasma 0S CS, 0S HS urine, NS HS urine, 4S CS urine, 6S CS urine) with minimal performance loss when applied to the development study (AUC = 0.85 [95% CI= 0.81–0.88], *SI Appendix*, Figs. S14–S16). Then, we conducted a prospectively planned cohort-based case-control study to test the pruned combined free GAGome MCED score in an independent and external retrospective cohort of apparently cancer-free adults from the Lifelines Cohort Study, a population-based biobank in the Netherlands. Of the 145,526 cancer-free adults recruited by Lifelines Cohort Study, after linkage with the Dutch Cancer Registry, we included 171 subjects with a confirmed cancer diagnosis within 18 mo of the baseline visit and 110 age-, sex-, and biochemistry-matched subjects that remained cancer-free through the follow-up visit (Fig. 2*A* and Table 1). The subject flow is described in *SI Appendix*, Fig. S17.

We observed that pruned scores were higher in cases than in controls (Fig. 2*B*). The pruned score predicted any type of cancer diagnosis within 18 mo of the baseline visit with an AUC = 0.65 (95% CI: 0.58–0.72; *SI Appendix*, Fig. S18 and Table S6). In the stage 0-II disease subset (N = 217, 107 cases), AUC = 0.62 (95% CI: 0.54–0.69), and in the diagnosis within 3 mo after the baseline visit subset, AUC = 0.69 (95% CI: 0.61–0.78). This performance was consistent with the expectation that as the score increased (albeit weakly) with the stage at diagnosis, it decreased with the time to the cancer diagnosis after the baseline visit . The discriminatory performance was similar across cancer types, ranging from AUC = 0.71 (95% CI: 0.59–0.82) in NSCLC to AUC = 0.58 (95% CI: 0.40–0.75) in gynecological cancers (*SI Appendix*, Fig. S18 and Table S6).

The pruned score performed remarkably well considering the appreciable population differences between the validation and development studies. First, while the validation controls from the Lifelines Cohort Study were not screened for potential comorbidities, the development study controls were selected for self-rated healthy status. Second, the country of origin of the subjects differed (Netherlands vs. Sweden or Italy) as well as the pre-analytical protocols. However, few controls with outlier GAGome features resulted in lower specificity than required in screening settings, which typically range from ≥95% in the elevated risk population to ≥99% in the general population (5). We explored correlations between subject characteristics and the pruned score to investigate potential confounders (*SI Appendix*, Table S7). Consistent with known confounders of free GAGomes (28), we found a significant linear correlation between the pruned score and serum C-reactive protein (CRP; Kendall correlation coefficient [*τ*] *=* 0.29, *P* < 2 × 10^−6^) and high-density lipoprotein-cholesterol (HDL-C) levels (*τ = −*0.16, *P* = 0.02). When we examined the correlation between the pruned score and CRP or HDL-C levels in the healthy controls included in the development study, we also found that scores were higher when either biomarker had an abnormal value (+60%, *P* = 0.0004; two-sided *t* test; *SI Appendix*, Fig. S19), indicating that the sensitivity was possibly underestimated in the development study. In the validation study’s subset without acute inflammation (CRP > 4 mg dL^−1^) or metabolic syndrome (HDL-C <1 mmol L^−1^; N = 121, 49 cases), almost all subjects with outlier GAGome values were controlled for, resulting in 31% sensitivity (95% CI: 14 to 47%) at 95% specificity to predict any type of cancer within 18 mo of the baseline visit (32% sensitivity to stage 0–II disease [95% CI: 14 to 48%] and 26% to stage I [95% CI: 5 to 47%]; Fig. 2*C* and *SI Appendix*, Table S6). Since the median follow-up time in the case arm was 7.8 y, we could confirm whether the detected cancers were likely to be clinically significant by classifying cases as poor prognosis (deaths <7.8 y after diagnosis; N = 7) vs. not (Fig. 2*D*). The pruned score had 43% sensitivity (95% CI: 14 to 86%) at 95% specificity to predict cancer with poor prognosis. At 99% specificity, it had 21% sensitivity for stage I cancers and 43% for cancer with poor prognosis (*SI Appendix*, Table S6). Overall, the pruned combined free GAGome MCED score appeared to be useful for MCED when confirmed in an independent screening-like population with acceptable sensitivity to stage I and poor prognosis cancers, which might lead to significant stage-shifting while limiting overdiagnosis.

### Free GAGome Dynamics in an In Vivo Model of Cancer Progression.

**Fig. 3.**
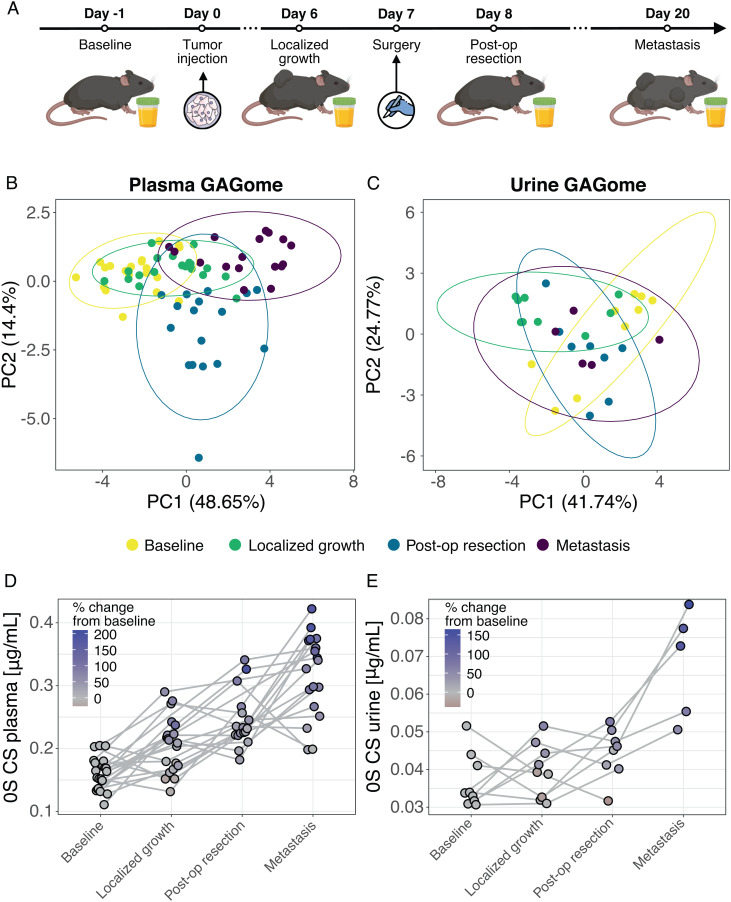
Changes in plasma and urine free GAGomes during cancer progression in mice. (*A*) Experimental design overview. Principal component analysis of (*B*) plasma and (*C*) urine free GAGomes at different cancer progression timepoints. The values in parentheses on axes show the percentage of explained variance for the respective principal component. Ellipses indicate the 95% CIs for a bivariate *t*-distribution fitted to data points belonging to each cancer progression timepoint. Longitudinal level of (*D*) plasma and (*E*) urine 0S (non-sulfated) CS concentration per mouse at different cancer progression timepoints. Key: PC, principal component.

We investigated whether the alterations in free GAGomes attributed to any cancer type were mechanistically associated with cancer onset and progression in vivo. We performed longitudinal measurements of urine and plasma free GAGomes in BALB/c (BALB/cAnNCrl) mice in which murine renal adenocarcinoma tumor cells were induced orthotopically on day zero (*N* = 20 in 10 metabolic cages; Fig. 3*A*). The kidney harboring the tumor was resected on day 7, and the mice were sacrificed on day 20. All mice developed metastases (>25 lesions, >100 in 85% of mice). This model was chosen since it recapitulates cancer progression from localized to metastatic recurrence after surgery (29). A principal component analysis showed that alterations in the plasma free GAGomes (Fig. 3*B*) and to a lesser extent in the urine free GAGomes (Fig. 3*C*) were consistent with progression from baseline (day 0) to localized growth (day 6) to post-operative resection (day 8) to metastasis (day 20). Consistent with the patterns observed in human cancer samples above, we found a credible linear increase in 0S CS across the timepoints in both plasma (% change at metastasis vs. baseline of 148% [95% CI: 91 to 213%]; Fig. 3*D*) and urine (116% [95% CI: 37 to 207%]; Fig. 3*E*). Changes in 4S CS are shown in *SI Appendix*, Fig. S20. These findings suggested that the free GAGome alterations captured by the scores were causally associated with cancer initiation and progression.

## Discussion

Consistent with previous pan-cancer analyses (17, 30), this study has shown the reprogramming of tumor metabolism—a cancer hallmark (14)—reflected in altered GAGomes across cancers. Compared to baseline physiological levels, we observed widespread changes in the free GAGomes of cancer patients already at stage I. We causally linked these changes to cancer progression in an in vivo mouse model. Together with the validation study, this experiment reinforces the plausible biological association between free GAGomes and the occurrence of any type of cancer. However, the exact mechanisms remain elusive, and elucidating them will be critical for minimizing false positives in a clinical setting (See *SI Appendix*, Discussion).

Nevertheless, we used these free GAGome alterations to develop a liquid biopsy test for MCED. Unlike genomics- and proteomics-based MCED assays that survey an entire landscape of potential alterations (e.g., >100,000 CpG sites with altered methylation^7^), free GAGomes comprise a comparatively finite feature set. As few as five GAGome features could extract meaningful information about cancer’s spatial and temporal status from an early stage. From a practical perspective, this allows for a relatively simple, low-cost assay, making it more feasible to implement in high-volume settings such as cancer screening and presumably with more robust predictive performance. Using the free GAGome MCED scores, the sensitivity to any type of stage I/low-grade cancer was 41.6 to 62.3% at 95% specificity. In comparison, other MCED assays have reported 39 to 73% sensitivity to stage I cancers. However, these estimates are limited to 12 cancer types generally considered “high-signal” and perform poorly in cancers that emit little cfDNA, such as genitourinary and brain (6, 8, 9). Notably, we observed that free GAGomes were altered in all 14 cancer types tested, including low- and high-grade gliomas and RCC, the latter consistent with our earlier studies (17, 21).

To our knowledge, only two assays have reported performance estimates in an external validation study (7, 13), of which only Lennon et al. included a representative population for MCED (13). Lennon et al. estimated the sensitivity for stage I cancers (10.2% at ~99% specificity) dropped significantly compared to the initially reported performance in early-stage cancers for the same technology (AUC = 0.91 with ~45% sensitivity to stage I cancers (8)). We also observed a similar trend during validation. These differences could arise for various reasons, including unknown biological or demographic factors across study populations, differences in sample collection or other pre-analytical procedures, overfitting/optimism during prediction modeling, and analytical precision of the underlying assay, all of which determines measurement noise. Our results show that simplifying the combined score and accounting for confounding conditions like acute inflammation—either by excluding subjects with an aberrant blood chemistry pre-test or by incorporating such information in the interpretation of the MCED test results as it is done with clonal hematopoiesis for the above mentioned MCED assays—may control these factors and maintain a high sensitivity to stage I cancer in an MCED population. At equivalent specificity, the combined score would find 2.1 times more stage I cancers than the MCED assay of Lennon et al. (21% vs. 10.2% at 99% specificity) despite the longer prediction time in our study (18 vs. 12 mo after sampling); and 25% more than the MCED assay of Klein et al (7) (21% vs. 16.8%) -  although this may be an underestimation since cases in Klein et al. were symptomatic at sampling and not selected from the same cohort as the controls. Notably, most cancers detected by our combined score had a poor prognosis, which may increase the odds of their clinical significance were they to be found earlier, potentially limiting overdiagnosis.

Based on this data and assuming a 1% cancer prevalence in a screening population for MCED, the estimated positive and negative predictive value of a free GAGome MCED test would be 17.5% and 99.2%, respectively. From a technical standpoint, based on the experience from this study and foreseeable optimizations, we expect a throughput of 100 patient samples per day per UHPLC-MS/MS instrument at a cost per sample <$50. This cost is 5–10 times lower than a previous estimate for a cfDNA/protein-based MCED test (8) and well below its presumed health economics value of ~$1200 (31). The final cost depends on whether the free GAGome MCED test requires one (urine or plasma) or two samples (both urine and plasma) per patient. Overall, we believe that a future screening program using a free GAGome-based MCED test appears realistic.

The metabolic nature of free GAGomes and their ability to detect cancer types that are poor cfDNA-shedders greatly complements genomic biomarker-based liquid biopsies, paving the way for a multimodal MCED approach. Such an approach may increase sensitivity to stage I cancers even further to a level where cancer mortality could be substantially curbed by early detection alone (32).

## Materials and Methods

### Development Study Design and Patient Recruitment.

The design was a case-control study with subjects enrolled both retrospectively and prospectively across four sites (Uppsala Umeå Comprehensive Cancer Consortium (U-CAN), Uppsala, Sweden; Sahlgrenska University Hospital, Göteborg, Sweden; Sabbatsberg Hospital, Stockholm, Sweden; and San Raffaele Hospital, Milan, Italy). The study population comprised cases defined as patients with confirmed cancer diagnosis (no history of cancer, active disease [treatment naïve or metastatic disease] across 14 cancer types) and controls defined as self-rated healthy subjects (moderated to very good health, no history nor known family history of cancer). All subjects provided informed consent at the sites under IRB approved protocols. The ethical approvals were granted by the Ethical Committee (*Regionala Etikprövningsnämnden*, currently renamed as *Etikprövningsmyndigheten*) in Gothenburg, Sweden (approvals: #047-16, #198-1, #940-17, #737-17, #469-17) and the Ethical Committee at San Raffaele Hospital, Milan, Italy (approval: #50/2018). Details on recruitment procedures and eligibility criteria are provided in the Supplementary Methods.

Clinical data related to age, sex, eligibility criteria, as well as date of death or last known alive, diagnosis, and tumor grade or stage for all cases, were retrieved from patients’ journals in the case arm and through a questionnaire in the control arm. Cases were grouped by cancer type and by stage/grade. Specifically, we classified cases as early-stage or low-grade vs. stage IV or high-grade as follows: TNM I-III vs. TNM IV in BC, CRC, NSCLC, RCC, BCa, HN; G1 (Mitotic count (10 HPF) <2 and Ki67 < 2) vs. G2 (Mitotic count (10 HPF) 2 to 20 and Ki67 3 to 20) in GNET; lower-grade glioma vs. glioblastoma multiforme in DG; FIGO stage I vs. II-IV in CST and I-II vs. III-IV in EC/OV; Binet stage A-B vs. C in LL; Anna Arbor stage I-II vs. III-IV in NHL; pathological Gleason grade < 7 vs. >= 7 in PCa. Further subsets were stage I/low-grade including all early-stage/low-grade except TNM II-III, FIGO stage II, Binet stage B, and Ann Arbor stage II; stage II including TNM II, FIGO stage II, Binet stage B, and Ann Arbor stage II; stage III including TNM III, FIGO stage III, and Ann Arbor stage III.

### Sample Collection and Pre-Analytical Procedures.

Across all subjects, we successfully analyzed a total of 969 plasma and 560 urine samples, so divided: for the case arm, 517 plasma samples in 14 cancer types and 220 urine samples in five cancer types and for the control arm, 452 plasma and 340 urine samples. A subset of 184 cancer (five cancer types) and 339 healthy subjects had combined plasma and urine samples available. All subjects were de-identified and registered according to applicable national laws for bio-banking.

Whole blood samples were collected in K2 EDTA-coated tubes at room temperature and processed within 15 min. The tubes were centrifuged (2,500 RCF for 15 min at 4°C) and the plasma supernatant transferred to separate cryovials for storage at −80°C until shipment in dry ice. Urine was an any-void spot collection in polypropylene cups with 100–220 µL urine aliquoted into cryovials for storage at −20°C until shipment in dry ice. For specific subject groups, there were protocol deviations in the plasma and urine centrifugation step (see Supplementary Methods) attributable to different pre-analytical protocols in the robotic handling of samples across sites. These deviations are not expected to exert a remarkable effect on free GAGome measurements.

### Free GAGome Measurements.

Free GAGome measurements were performed in a single-blinded GLP-compliant central laboratory using MIRAM^®^ Free Glycosaminoglycan Kit (Elypta AB, Sweden), which is a standardized kit for GAG extraction, detection, and quantification by ultra-high-performance liquid chromatography (UHPLC) coupled with electrospray ionization triple-quadrupole mass spectrometry system (ESI-MS/MS, Waters® Acquity I-class Plus Xevo TQ-S micro). The total instrument run-time was 15 min per sample injection. A single UHPLC column equipped with a pre-column guard (Waters® ACQUITY UPLC BEH C18 VanGuard Pre-column) was sufficient to analyze all samples in this study with no quality deterioration observed over time. The analytical performance characteristics of the kit have been previously described (24).

In short, the kit is based on a method by Volpi et al. (33). The assay consists of the enzymatic depolymerization of GAGs from the sample into disaccharides by *Chondroitinase ABC* and *Heparinase I-II-III*. The method omits proteolytic digestion, thereby limiting the derived depolymerized GAGs to the protein-free fraction—or free GAGs. Following depolymerization, disaccharides are labeled using 2-aminoacridone and injected into an UHPLC-MS/MS for separation and detection. The peaks of the 17 disaccharides are acquired at using multiple reaction monitoring analysis implemented in the mass spectrometry software (Waters® TargetLynx). The chromatographic conditions and MS configuration were set in accordance with the kit instruction for use.

Each sample was measured in singleton. The so-measured free GAGome consisted of the semi-absolute concentrations of 17 disaccharides, corresponding to eight different sulfation patterns of CS and HS, and the HA disaccharide. Specifically, we quantified eight CS disaccharides (0S CS, 2S CS, 6S CS, 4S CS, 2S6S CS, 2S4S CS, 4S6S CS, TriS CS) and eight HS disaccharides (0S HS, 2S HS, 6S HS, NS HS, NS6S HS, NS2S HS, 2S6S HS, TriS HS). We have previously noted prognostic and diagnostic potential of compositional GAGome features (17, 21–23), so we expanded the free GAGome to include an additional 22 calculated features informative of GAG biology: the total CS and total HS concentration as the sum of the corresponding disaccharide concentrations, the CS charge [−] and HS charge [−] as the weighted sum of sulfated disaccharides, where the weight is the count of sulfo-groups in each disaccharide, two ratios (4S CS/0S CS and 6S CS/0S CS), and the relative concentration (or mass fraction, in %) of each of the 16 CS and HS disaccharides by normalizing each concentration by the total CS and HS concentration, respectively. For each plasma or urine sample, the free GAGome consisted of maximally 39 features.

We excluded from downstream analyses those GAGome features below the limit of detection in most of plasma or urine samples—in other words, we considered such GAGome features undetectable in plasma or urine. We considered a GAGome feature detectable in a biofluid if the median concentration across all samples was above 0.1 µg mL^−1^ (24). We speculate that K2 EDTA contamination in plasma may be responsible for incomplete HS depolymerization, resulting in disaccharide levels below the limit of detection, which is in contrast to previous detection of HS—albeit in low amounts—when this is measured in serum (34). Next, we calculated four dependent features in plasma and 11 in urine, based on the detectable GAGome features. For plasma features, the following were calculated: a) total CS concentration, b) mass fractions of 0S CS, c) 4S CS, and d) 4S/0S CS ratio. In urine, the dependent features were a) total CS and b) total HS concentrations, mass fractions of c) 0S CS, d) 4S CS, e) 6S CS, f) 2S6S CS, g) 0S HS, h) NS HS, i) and j) two CS ratios (6S/0S and 4S/0S), and k) CS negative charge. Cumulatively, the final free GAGome had 23 (six plasma and 17 urine) detectable features.

We identified four (0.7%) urine and three (0.3%) plasma outliers in a two-step procedure (see Supplementary Methods). The reported sample numbers throughout the manuscript excluded outliers.

### Statistical Analysis and Bayesian Estimation.

We carried out estimation of group differences in GAGome features by Bayesian estimation and equivalence testing (35). In short, we modeled each individual standardized GAGome feature as a response with a skew-normal distribution in a mixed effects model, where diagnosis was a fixed effect and experimental batch was treated as a random factor. We modeled the group-specific variances as a multiplicative interaction between the cancer-type and experimental batch. We estimated the predictors using ~*Normal* (0,5) for the means and ~*Gamma* (1,2) for the standard deviation. We tested the sensitivity to the prior choice by repeating the procedure with ~*Normal* (0,2.5) and ~*Normal* (0,10) for the prior for group means. We considered the convergence of Bayesian estimation acceptable if the effective sample size > 5,000 and the potential scale reduction factor *R* < 1.001. We used the posterior samples to compute the 95% credible interval (95% CI) of group medians for each GAGome feature conditional on the diagnosis. Next, we computed the 95% CI for the difference in medians of each cancer diagnosis versus healthy. We omitted ratio features (4S CS/0S CS and 6S CS/0S CS) from this comparison because their respective models did not converge. We deemed that a GAGome feature was correlated with a cancer diagnosis vs. healthy subject group if 95% CI of the difference in means did not cross 0 and no more than 5% fell inside the pre-specified ROPE interval around 0. We defined the ROPE boundaries as 0.2 of the overall standardized mean since the coefficient of variation previously observed in the measurement of GAGome features ranged between 15 and 25% (24). Bayesian estimation was carried out using the *brms* (2.14.4) (36, 37) and *tidybayes* (2.3.1) packages in R (4.0.4).

### Development of Free GAGome MCED Scores.

We aimed to identify a minimal subset of GAGome features which were informative for discrimination between cancer vs. healthy subjects. To this end, we used projection predictive variable selection to select relevant features independently in urine, plasma, and combined free GAGomes. First, we fit three reference (plasma-only, urine-only, and combined) Bayesian multivariable logistic regressions with cancer (aggregating all cancer types) vs. healthy as a response and standardized detectable GAGome features as predictors. We excluded plasma CS charge as a predictor from plasma-only and combined score, because 4S CS was the only detectable plasma sulfated (i.e., charged) feature. We used a heavy-tailed t-distribution (df = 7) with location 0 and scale 2.5 as a prior on the intercept and coefficients for all predictors. We tested the sensitivity to the prior choice by repeating the procedure with ~*t-student* (0,1.25) and ~*t-student* (0,5) on the intercept and coefficients for all predictors. We fit the models using *rstanarm* package *(2.21.1)* with four chains for a total of 4,000 iterations (2,000 warm-up). The Bayesian R^2^ of urine, plasma, and combined reference models was of 0.32, 0.41, and 0.56, respectively.

Next, we carried out the variable selection using leave-one-out cross-validation forward selection using the *cv_varsel* function from the *projpred* package (25, 26) in R (4.0.4). We selected the sub-model of a minimal size such that the estimated difference of sum of log predictive densities (ELPD) between the reference model and sub-model was at most one standard error away from the zero (default). We then selected and projected the final set of sub-models, with the default suggested optimal model size (plasma—three features, urine—13 features, combined—14 features). Finally, for each model, we projected the 400 draws of the sub-model of the selected size and predicted the response using draws of the linear predictor (*proj_linpred* function, averaged over all parameters). The effect size of the response, called free GAGome MCED score, was predicted as log-odds of any-type cancer. Confidence intervals for sensitivity at 95% specificity were calculated using the binomial approximation.

### Internal Validation of the Free GAGome MCED Scores.

We validated the variable selection procedure by bootstrap analysis. To this end, we analyzed 500 bootstraps for plasma and 1,000 for urine and combined datasets. In each bootstrap, we first fit the reference logistic Bayesian regression model. We used the same priors and fitting parameters as described above (t-distribution priors with df = 3, 4,000 iterations with 2,000 warm-up samples). Second, we carried out the projection predictive variable selection using leave-one-out cross-validation. Projection used 400 samples, with projected model size determined automatically by ELPD or set to 1/10 of the number of cases in the original dataset. The constraints on the maximal number of parameters were 51, 22, and 18 for plasma, urine, and combined, respectively. Finally, we predicted the response (log-odds of any-type cancer) using draws of the linear predictor for the three datasets: the bootstrap, assessment dataset (samples left out of the bootstrap), and the original dataset. We recorded the selected model size, AUC, sensitivity at 95% specificity, and scaled Brier metric for the full and projected model on the bootstrapped, original, and assessment dataset.

### Putative Cancer Location Analysis.

We carried out this analysis on the subset of cancer cases with combined free GAGomes available. We preselected detectable plasma and urine GAGome features as described above, which resulted in the inclusion of an additional urine GAGome feature, 4S6S CS. We trained a multinomial Bayesian Additive Regression Trees (*BART*) (27) on the samples in the training set (60%), where the cancer type was the response and the preselected GAGome features were the predictors. We assigned the category with the highest mean posterior probability as the predicted cancer type. We validated the prediction accuracy on the test set (40%). We further assigned cancer types to two putative cancer location (PCL) classes: respiratory tract (NSCLC, HN) and genitourinary tract (RCC, PCa, BCa). Confidence intervals for accuracy and balanced accuracy were calculated by 5’000 bootstrapped replicates using the normal approximation. BART regression was performed using the package *BART* (2.9) in R (4.0.4).

### Survival Analysis.

In the subset of cancer cases with survival data recorded, we correlated OS with the plasma, urine, and combined free GAGome MCED scores. OS was calculated as the time between the date of sampling and the time of event. The time of event is defined as right-censoring (date of last follow-up without the event) or as date of death from any cause. Multivariable survival analyses were performed by fitting a Cox proportional hazard model to estimate the odds ratio for the variables of interest and the 95% confidence interval. The log-rank statistical test was utilized to determine the significance of the regression. For each score, we constructed a multivariable Cox model also using the following variables for regression of survival: age (as a scaled 3-knot splined continuous variable, in years), sex (male vs. female, binary variable), late stage (stage IV/high-grade vs. not, binary variable), and cancer type (in plasma only, diffuse glioma vs. not, binary variable). Missing data were omitted. The validity of the proportional hazard assumption was checked using a two-sided *t* test between transformed survival time and the scaled Schoenfeld residuals (*P >* 0.01 for the global fit). We checked for overfitting by performing internal validation of each multivariable model using a bootstrapping algorithm (1,000 bootstraps) and observing the change in Somers’ D rank correlation (Dxy) statistics in the original datasets as opposed to the test set. A correction <20% was considered acceptable.

For each score, we dichotomized patients into two groups, “Low” vs. “High” score depending on the 95% specificity cutoff obtained for that score. We then fitted Kaplan–Meier survival curves to the two groups, and the statistical significance for survival difference (in terms of HR for OS in the “Low” vs. “High” risk group) was evaluated using the log-rank test. We performed subset analyses in stage I–III or low-grade patients only and stage IV or high-grade patients only. In a sensitivity analysis, we identified an alternative cutoff for each score by using maximally selected rank statistics if significant (M-score *P* < 0.01) and repeated the Kaplan–Meier survival analysis. We performed subset analyses for each cancer type. *P*-values < 0.01 were considered significant*.* Statistical analyses were performed using the packages *survival* (3.2) and *rms* (6.2) and *maxstat* (0.7) in R (4.0.4).

### External Validation of Free GAGome MCED Scores.

We sought to validate the free GAGome MCED scores in a prospectively planned cohort-based case-control validation study. We identified Lifelines Cohort Study (38) as a suitable biobank that recruited an external population-based cohort representative of a potential screening population. Lifelines is a multi-disciplinary prospective population-based cohort study examining in a unique three-generation design the health and health-related behaviors of 167,729 persons living in the North of the Netherlands. It employs a broad range of investigative procedures in assessing the biomedical, socio-demographic, behavioral, physical, and psychological factors which contribute to the health and disease of the general population, with a special focus on multi-morbidity and complex genetics.

Biospecimen collection (urine and plasma) in Lifelines was done at a baseline visit where each included subject was also required to fill in a questionnaire with general information and self-reported health status, which included if the subject had received a cancer diagnosis before or at the time of sampling. Each subject was then required to partake in an 18-mo follow-up visit in which any cancer diagnosis received after the baseline visit was reported. We defined two arms: cases as subjects that were cancer-free at the baseline visit but reported a cancer diagnosis at the 18-mo follow-up visit and controls as subjects that were cancer-free at the baseline visit and stayed cancer-free at the 18-mo follow-up visit. See Supplementary Methods for the eligibility criteria.

Of 145,526 subjects in Lifelines potentially eligible for this study, about 700 reported a cancer diagnosis after the baseline visit at the 18-mo follow-up visit. We randomly selected 261 cases in the case arm that self-reported a cancer diagnosis at a follow-up visit 18 mo after the baseline visit (capping to n = 50 the maximum number of subjects with a given self-reported cancer type), and we pseudo-randomly selected 110 age-, sex-, and biochemistry-matched subjects that stayed cancer-free through the follow-up visit. Biochemistry matching was performed on CRP, ALP, calcium, hemoglobin, and neutrophile/thrombocyte/lymphocyte count distribution. We performed a linkage with the Dutch Cancer Registry to a) verify a cancer diagnosis within 18 mo after the baseline visit in 171 (66%) subjects in the case arm (thus excluding 90 unverified cases), of which 35 (20%) were diagnosed within 3 mo after the baseline visit and 107 (63%) were diagnosed with stage 0-II disease (of which 22 (21%) within 3 mo after the baseline visit) and b) retrieve further information on the cases, specifically: the time between the baseline visit and cancer diagnosis, the diagnosed cancer type, and the TNM stage at diagnosis.

We focused on the combined free GAGome MCED score since it reported the highest AUC in the development study. To reduce the odds of overfitting and further increase the robustness and generalizability of the model, we opted to repeat the variable selection procedure in the development study limited only to independently measured GAGome features, that is measured in semi-absolute concentrations (μg mL^−1^), which are less susceptible to technical variation. Using the default suggested model size, we produced a pruned combined free GAGome MCED score consisting only of five features with minimal loss of performance metrics in the development study (plasma 0S CS, 0S HS urine, NS HS urine, 4S CS urine, 6S CS urine).

The statistical analysis of the diagnostic performance for the pruned score was performed as described for the development study. Correlation between blood chemistry biomarkers and the pruned scores was computed using the Kendall correlation coefficient with a permutation test to assess the corresponding *P*-value adjusted for multiple testing using the Holm correction (correlations with adjusted *P* < 0.05 were considered significant). Score performance was assessed using AUROC and sensitivity at 95% and 99% specificity across different patient subsets. All computations were performed in R (4.0.4).

### Mouse Experiment.

The experiment was carried out by Oncodesign Biotechnology (Dijon, France) under AAALAC accreditation following approval from the institutional Ethical Committee (ref: 2016041218566820). In short, 28 female BALB/c (BALB/cAnNCrl) mice, 5–6 wk old at reception, were used in the experiment—3 as controls. RenCa tumors were induced on day zero (D0) orthotopically on 25 female BALB/c mice under anesthesia, wherein five mice were used to replace eventual dropout during the experiments. The animal abdomen was opened through a median incision under aseptic conditions. 5 **×** 10^5^ murine renal adenocarcinoma (RenCa) tumor cells (American Type Culture Collection, USA), in 25 μL of Roswell Park Memorial Institute medium, were slowly injected in subcapsular space of the left kidney. At day seven (D7), the abdomen of mice was opened and the kidney containing injected RenCa cells was resected.

Blood was collected from 20 mice at each timepoint. Dropouts due to compassionate termination were replaced. Blood (50 μL per sample) was collected into K2 EDTA tube by jugular venipuncture. Intracardiac blood collection was conducted as a terminal procedure under deep isoflurane gas anesthesia. Blood was collected from animals at the following timepoints on day 1 (24 h before engraftment), day 6 (24 h before kidney resection), day 8 (24 h after kidney resection), and day 20 (day of mice termination). Therefore, each mouse generated up to four plasma samples. Blood was collected into collection tubes with anticoagulant (K2 EDTA). Tubes were centrifuged (2000 RCF, 10 min, room temperature) to obtain plasma. Plasma samples were stored in polypropylene tubes at −20°C until shipment.

Urine was collected from the same 20 mice split in ten metabolic cages. Dropouts due to compassionate termination were replaced. The animals were kept in metabolic cages for the collection of pooled urine of two mice per cage for 24 h at + 4°C. All urine was collected from animals at the following timepoints from day 1 to day 2 (24 h before OT engraftment), day 5 to 6 (24 h before kidney resection), day 7 to 8 (24 h after kidney resection), and day 19 to 20 (24 h before mice termination) in polypropylene tubes and stored at −20°C until shipment. Each group of two mice generated four urine samples. All surviving mice were terminated on day 20 as described above. At day of termination, the lung from all mice was collected and weighted. In addition, macroscopic lung metastases count (if possible) was performed in each mouse.

Plasma and urine GAGome features were subsequently measured from each timepoint (baseline: day 1 to 2; localized growth: day 5 to 6; post-operative resection: day 7 to 8; metastasis: day 19 to 20). Scaled and centered features were used for principal component analysis and labeled according to the timepoint of each sample. We also visualized the difference in GAGome features in individual mice across timepoints by plotting the values and fitting a loess regression curve to each one. For each individual standardized GAGome feature, in either urine or plasma, we estimated the population level change with time using a Bayesian mixed effects regression model. We set the sampling timepoint as a fixed effect and each mouse as a random effect. Missing or incomplete data at a given timepoint were omitted (specifically, only urine samples where both mice in a cage were alive at a given timepoint were used). Posterior samples were used to estimate the percentage difference in GAGome feature levels between individual timepoints and baseline. 95% credible intervals for the percentage differences were also calculated based on the posterior samples. Bayesian estimation was carried out using the *brms* (2.14.4) and *tidybayes* (2.3.1) packages in R (4.0.4).

### Code Availability.

A synthetic dataset with standardized GAGome values and code for the development of free GAGome MCED scores by projection predictive variable selection is deposited at: https://github.com/SysBioChalmers/GAGome-MCED.

## Supplementary Material

Appendix 01 (PDF)

Dataset S01 (XLSX)

Dataset S02 (XLSX)

Dataset S03 (XLSX)

## Data Availability

The data used in this study are not publicly available because they use clinical records protected by patient confidentiality. Requests for access to de-identified data can be directed to the corresponding author. Data that can be shared can be released via a material transfer agreement.

## References

[1] R. A. Smith , Cancer screening in the United States, 2019: A review of current American Cancer society guidelines and current issues in cancer screening. CA Cancer J. Clin. **69**, 184–210 (2019). 30875085 10.3322/caac.21557

[2] S. Bratulic, F. Gatto, J. Nielsen, The translational status of cancer liquid biopsies. Regen. Eng. Transl. Med., 10.1007/s40883-019-00141-2 (2019).

[3] D. Crosby , Early detection of cancer. Science **375**, eaay9040 (2022), 10.1126/science.aay9040. 35298272

[4] E. Heitzer, I. S. Haque, C. E. S. Roberts, M. R. Speicher, Current and future perspectives of liquid biopsies in genomics-driven oncology. Nat. Rev. Genet. **20**, 71–88 (2019). 30410101 10.1038/s41576-018-0071-5

[5] A. Hackshaw, C. A. Clarke, A. R. Hartman, New genomic technologies for multi-cancer early detection: Rethinking the scope of cancer screening. Cancer Cell **40**, 109–113 (2022). 35120599 10.1016/j.ccell.2022.01.012

[6] M. C. Liu , Sensitive and specific multi-cancer detection and localization using methylation signatures in cell-free DNA. Ann. Oncol. **31**, 745–759 (2020). 33506766 10.1016/j.annonc.2020.02.011 PMC8274402

[7] E. A. Klein , Clinical validation of a targeted methylation-based multi-cancer early detection test using an independent validation set. Ann. Oncol. **32**, 1167–1177 (2021). 34176681 10.1016/j.annonc.2021.05.806

[8] J. D. Cohen , Detection and localization of surgically resectable cancers with a multi-analyte blood test. Science **359**, 926–930 (2018). 29348365 10.1126/science.aar3247 PMC6080308

[9] S. Cristiano , Genome-wide cell-free DNA fragmentation in patients with cancer. Nature **570**, 385–389 (2019). 31142840 10.1038/s41586-019-1272-6 PMC6774252

[10] X. Chen , Non-invasive early detection of cancer four years before conventional diagnosis using a blood test. Nat. Commun. **11**, 3475 (2020). 32694610 10.1038/s41467-020-17316-z PMC7374162

[11] S. Y. Shen , Sensitive tumour detection and classification using plasma cell-free DNA methylomes. Nature **563**, 579–583 (2018). 30429608 10.1038/s41586-018-0703-0

[12] Y. Zhang , Pan-cancer circulating tumor DNA detection in over 10,000 Chinese patients. Nat. Commun. **12**, 11 (2021). 33397889 10.1038/s41467-020-20162-8 PMC7782482

[13] A. M. Lennon , Feasibility of blood testing combined with PET-CT to screen for cancer and guide intervention. Science **369**, eabb9601 (2020). 32345712 10.1126/science.abb9601 PMC7509949

[14] R. J. DeBerardinis, N. S. Chandel, Fundamentals of cancer metabolism. Sci. Adv. **2**, e1600200 (2016). 27386546 10.1126/sciadv.1600200 PMC4928883

[15] N. N. Pavlova, C. B. Thompson, The emerging hallmarks of cancer metabolism. Cell Metab. **23**, 27–47 (2016). 26771115 10.1016/j.cmet.2015.12.006 PMC4715268

[16] F. Gatto, I. Nookaew, J. Nielsen, Chromosome 3p loss of heterozygosity is associated with a unique metabolic network in clear cell renal carcinoma. Proc. Natl. Acad. Sci. U.S.A. **111**, E866–E875 (2014). 24550497 10.1073/pnas.1319196111 PMC3948310

[17] F. Gatto , Glycosaminoglycan profiling in patients’ plasma and urine predicts the occurrence of metastatic clear cell renal cell carcinoma. Cell Rep. **15**, 1822–1836 (2016). 27184840 10.1016/j.celrep.2016.04.056

[18] Y. H. Chen , The GAGOme: A cell-based library of displayed glycosaminoglycans. Nat. Met. **15**, 881–888 (2018). 10.1038/s41592-018-0086-z 30104636

[19] N. Afratis , Glycosaminoglycans: Key players in cancer cell biology and treatment. FEBS J. **279**, 1177–1197 (2012). 22333131 10.1111/j.1742-4658.2012.08529.x

[20] J. Wei, M. Hu, K. Huang, S. Lin, H. Du, Roles of proteoglycans and glycosaminoglycans in cancer development and progression. Int. J. Mol. Sci. **21**, 5983 (2020). 32825245 10.3390/ijms21175983 PMC7504257

[21] F. Gatto , Plasma glycosaminoglycans as diagnostic and prognostic biomarkers in surgically treated renal cell carcinoma. Eur. Urol. Oncol. **1**, 364–377 (2018). 31158075 10.1016/j.euo.2018.04.015 PMC8253162

[22] F. Gatto , Plasma and urine free glycosaminoglycans as monitoring biomarkers in nonmetastatic renal cell carcinoma—a prospective cohort study. Eur. Urol. Open Sci. **42**, 30–39 (2022). 35911082 10.1016/j.euros.2022.06.003 PMC9334826

[23] F. Gatto, M. Maruzzo, C. Magro, U. Basso, J. Nielsen, Prognostic value of plasma and urine glycosaminoglycan scores in clear cell renal cell carcinoma. Front. Oncol. **6**, 253 (2016). 27933273 10.3389/fonc.2016.00253 PMC5121125

[24] D. Tamburro, Analytical performance of a standardized kit for mass spectrometry-based measurements of human glycosaminoglycans. J. Chromatogr. B Analyt. Technol. Biomed. Life Sci. **1177**, 122761 (2021), 10.1016/j.jchromb.2021.122761. 34052753

[25] A. Catalina, P.-C. Bürkner, A. Vehtari, "Projection Predictive Inference for Generalized Linear and Additive Multilevel Models" in International Conference on Artificial Intelligence and Statistics, G. Camps-Valls, F.J.R. Ruiz, I. Valera, Eds. (Proceedings of Machine Learning Research, Valencia , Spain, 2022) pp. 1–17. 10.48550/arXiv.2010.06994.

[26] J. Piironen, M. Paasiniemi, A. Vehtari, Projective inference in high-dimensional problems: Prediction and feature selection. Electron. J. Stat. **14**, 2155–2197 (2020).

[27] R. Sparapani, C. Spanbauer, R. McCulloch, Nonparametric machine learning and efficient computation with bayesian additive regression trees: The BART R package. J. Stat. Software **97**, 1–66 (2021).

[28] S. Bratulic , Analysis of normal levels of free glycosaminoglycans in urine and plasma in adults. J. Biol. Chem. **298**, 101575 (2022). 35007531 10.1016/j.jbc.2022.101575 PMC8888457

[29] P. Sobczuk , Choosing the right animal model for renal cancer research. Transl. Oncol. **13**, 100745 (2020). 32092671 10.1016/j.tranon.2020.100745 PMC7036425

[30] A. Salanti , Targeting human cancer by a glycosaminoglycan binding malaria protein. Cancer Cell **28**, 500–514 (2015). 26461094 10.1016/j.ccell.2015.09.003 PMC4790448

[31] A. Tafazzoli , The potential value-based price of a multi-cancer early detection genomic blood test to complement current single cancer screening in the USA. Pharmacoeconomics, 10.1007/s40273-022-01181-3 (2022). PMC9550746 36038710

[32] C. A. Clarke , Projected reductions in absolute cancer–related deaths from diagnosing cancers before metastasis, 2006–2015. Cancer Epidemiol. Biomarkers Prev. **29**, 895–902 (2020). 32229577 10.1158/1055-9965.EPI-19-1366

[33] N. Volpi, F. Galeotti, B. Yang, R. J. Linhardt, Analysis of glycosaminoglycan-derived, precolumn, 2-aminoacridone–labeled disaccharides with LC-fluorescence and LC-MS detection. Nat. Protoc. **9**, 541–558 (2014). 24504479 10.1038/nprot.2014.026

[34] W. Wei, M. R. Niñonuevo, A. Sharma, L. M. Danan-Leon, J. A. A. Leary, Comprehensive compositional analysis of heparin/heparan sulfate-derived disaccharides from human serum. Anal. Chem. **83**, 3703–3708 (2011). 21473642 10.1021/ac2001077 PMC3094484

[35] J. K. Kruschke, Bayesian estimation supersedes the t test. J. Exp. Psychol. Gen. **142**, 573–603 (2013). 22774788 10.1037/a0029146

[36] P. C. Bürkner, brms: An r package for bayesian multilevel models using stan. J. Stat. Software **80**, 1–28 (2017).

[37] P. C. Bürkner, Advanced Bayesian multilevel modeling with the R package brms. R J. **10**, 395–411 (2018).

[38] S. Scholtens , Cohort profile: LifeLines, a three-generation cohort study and biobank. Int. J. Epidemiol. **44**, 1172–1180 (2015). 25502107 10.1093/ije/dyu229

