# Supplementary material for: Noninvasive detection of any-stage cancer using free glycosaminoglycans
Source: Proc Natl Acad Sci U S A. 2022 Dec 5;119(50):e2115328119. doi: 10.1073/pnas.2115328119 (PMC9897435; doi:10.1073/pnas.2115328119)
Supplement: Supplementary file 1 — Appendix 01 (PDF) [file pnas.2115328119.sapp.pdf]

PNAS

Supporting Information for
Noninvasive detection of any-stage cancer using free glycosaminoglycans

Sinisa Bratulic, Angelo Limeta, Saeed Dabestani, Helgi Birgisson, Gunilla Enblad, Karin Stålberg, Göran Hesselager, Michael Häggman, Martin Höglund, Oscar E Simonson, Peter Stålberg, Henrik Lindman, Anna Bång-Rudenstam, Matias Ekstrand, Gunjan Kumar, Ilaria Cavarretta, Massimo Alfano, Francesco Pellegrino, Thomas Mandel-Clausen, Ali Salanti, Francesca Maccari, Fabio Galeotti, Nicola Volpi, Mads Daugaard, Mattias Belting, Sven Lundstam, Ulrika Stierner, Jan Nyman, Bengt Bergman, Per-Henrik Edqvist, Max Levin, Andrea Salonia, Henrik Kjölhede, Eric Jonasch, Jens Nielsen, Francesco Gatto

Francesco Gatto
Email: francesco.gatto@ki.se.

This PDF file includes:

Supporting Methods
Supporting Note
Supporting Discussion
Figures S1 to S20
Tables S1 to S7
Legends for Datasets S1 to S3
SI References

Other supporting materials for this manuscript include the following:

Datasets S1 to S3

Supporting methods

Recruitment procedures and eligibility criteria. Patients were enrolled retrospectively among plasma donors included in the Uppsala Umeå Comprehensive Cancer Consortium (U-CAN), Sweden30. The inclusion criteria were: for cases - diagnosis of cancer with any of the following cancer types: breast ductal invasive carcinoma (BC), colorectal carcinoma (CRC), cervical squamous cell carcinoma (CST), diffuse glioma (DG), small intestinal neuroendocrine tumor (GNET), endometrial adenocarcinoma (EC), chronic lymphoid leukaemia (LL), diffuse large B-cell lymphoma (NHL), non-small cell lung cancer (NSCLC), ovarian epithelial carcinoma (OV) and prostate adenocarcinoma (PCa); active disease [treatment naïve or metastatic disease] at the time of sampling; older than 18 year old at the time of sampling.
The exclusion criteria were: history of non-basal cell carcinoma cancer; samples collected before 2015 for all diagnosis groups except CRC (2012) and LL (2014). Patients were retrospectively identified by random choice based on the eligibility criteria so that between 14 to 40 patient per cancer type were included, of which 50% were early-stage/low-grade and 50% late-stage/high-grade (as defined below).
Patients were enrolled prospectively at Sahlgrenska University Hospital, Göteborg, Sweden - for bladder cancer (BCa), NSCLC, head and neck squamous cell carcinoma (HN), renal cell carcinoma (RCC); at San Raffaele Hospital, Milan, Italy for PCa; and at Sabbatsberg Hospital, Stockholm, Sweden for the healthy subjects. All patients donated both plasma and urine specimens during a single sampling visit. The eligibility criteria were: for NSCLS and HN - inclusion: ECOG performance status 0-2, diagnosis of non-small cell lung cancer or head and neck squamous cell carcinoma, metastatic disease, predicted life expectancy over 2 months; and exclusion: lack of proper compliance to accept samplings; for PCa - inclusion: referral to robotic-assisted laparoscopic prostatectomy for prostate cancer, serum PSA at clinical diagnosis available, transrectal ultrasound guided biopsy report available, fit to undergo all protocol procedures; no exclusion criteria; for RCC – inclusion: referral for partial or radical nephrectomy for suspicion of renal cell carcinoma, predicted life expectancy over 2 months, standard imaging evaluation 12 weeks prior to inclusion, planned for standard imaging within 16 weeks after start of therapy; exclusion: lack of proper compliance to accept continuous samplings; for the BCa - inclusion: referral to transurethral resection of the bladder (TURB) for BCa, urine cytology available at diagnosis, fit to undergo all protocol procedures; exclusion: age less than 18 years, history of bladder or prostate radiation, prior diagnosis of cancer; for healthy subjects – inclusion: self-rated health at least "moderate", age between 21 and 78 years old, fit to undergo all protocol procedures; exclusion: previous history of cancer (except non-melanoma skin cancer), family history of cancer among first-degree relatives, and for men, total serum PSA ≥ 0.5 ng/mL within the last 5 years or upon registration.

Sample collection and pre-analytical procedures. For specific subject groups, there were protocol deviations in the plasma and urine centrifugation step. In the healthy subjects, plasma centrifugation was at 1100-1300 RCF at room temperature for 10 to 20 minutes. In the U-CAN patients, plasma centrifugation was at 2400 RCF at room temperature for 7 minutes. In the BCa patients, plasma centrifugation was at 2000 RCF at room temperature for 10 minutes. In the BCa cohort, urine was also centrifuged at 2,000 RCF at room temperature for 5 minutes.

Outlier detection. First, we applied the Tukey method on the measurements of total CS concentration and 0S CS relative concentration (as representative GAGome features) across all samples following the Box-Cox transformation of the variables. Then, so identified outliers were manually inspected to confirm instances of kit failure by an expert operator, and the samples were excluded and not repeated due to insufficient volume. Additionally, we considered measurements where 0S CS was zero as outliers because this value is more likely attributable to enzymatic failure than a physiological value. We identified a total of 4 (0.7%) urine (2 RCC and 2 H) and 3 (0.3%) plasma (1 CRC, 1 PCa and 1 H) outlier samples and excluded them from downstream analysis. We concluded that outliers were rare and random. The reported sample numbers throughout the manuscript excluded outliers.

Validation of MCED GAG scores. For cases, inclusion criteria were: adults older than 18 years old; self-reported cancer-free at the baseline visit; self-reported cancer between the baseline visit and the 18-month follow-up visit; and exclusion criteria were: unverified cancer diagnosis by means of linkage with the Dutch Cancer Registry; outlier free GAGome in either plasma or urine. For controls, inclusion criteria were: adults older than 18 years old; self-reported cancer-free at the baseline visit; self-reported cancer-free at the 18-month follow-up visit; and exclusion criteria were: outlier free GAGome in either plasma or urine (as defined in the Methods).

Supporting notes

Survival analysis. We used maximally selected rank statistics to determine an optimized cut-off for the plasma, urine, and combined pan-cancer GAG scores (plasma: cut-off score = 2.92, $M_{statistic}$ = 4.002, p = 0.0022; urine: cut-off score = 3.29, $M_{statistic}$ = 3.52, p = 0.0096; combined: cut-off score = 4.53, $M_{statistic}$ = 3.62, p = 0.0057). For each score, we dichotomized patients in “High” vs. “Low” groups depending on whether their individual score was above or below the score-specific cut-off. For all three scores, the risk groups correlated with OS across and within cancer types (unadjusted HR = 1.87 (95% CI = 1.37-2.57), $p < 0.001$ in plasma Figure S11A, S12A; HR = 2.54 (95% CI = 1.46-3.95), $p < 0.001$ in urine Figure S11B, S12B; HR = 2.79 (95% CI = 1.61-4.88), $p < 0.001$ when combined, Figure S11C, S12C).

Supporting discussion

Biological origin of free GAGome alterations in cancer. Free GAGome alterations here associated with cancer entailed several structural and concentration differences across CS and HS disaccharides, notably resulting in the elevation of non-sulfated CS. We recapitulated this association in an in vivo mouse model. The exact mechanisms behind these alterations remain unclear. GAGs are ubiquitous in the human body. Tissue and leukocyte GAGs are thought to carry almost completely sulfated CS1. Therefore, these are unlikely sources for the elevation of non-sulfated CS. Aging and other co-morbidities may confound the specificity of free GAGomes to cancer. However, we observed an acceptable performance of the free GAGome MCED score in the validation study, notwithstanding the fact that both cases and controls in that cohort were matched by age, sex, and blood biochemistry. In addition, significant GAGome changes have not been observed across age groups or abnormal blood chemistry values in healthy subject (ref. 27 in the main text). Few small scale studies investigated biofluidic GAGomes in disease. Among these, respiratory failure and septic shock did not alter plasma or urine non-sulfated CS remarkably despite the fact that these conditions directly affect the GAG-rich endothelial glycocalyx of lungs and kidneys, respectively 2,3. Our results collectively suggest a tumor-specific origin for the increase of non-sulfated CS. Elucidating the mechanism behind these GAGome alterations will be critical to minimize false positives in certain clinical settings.

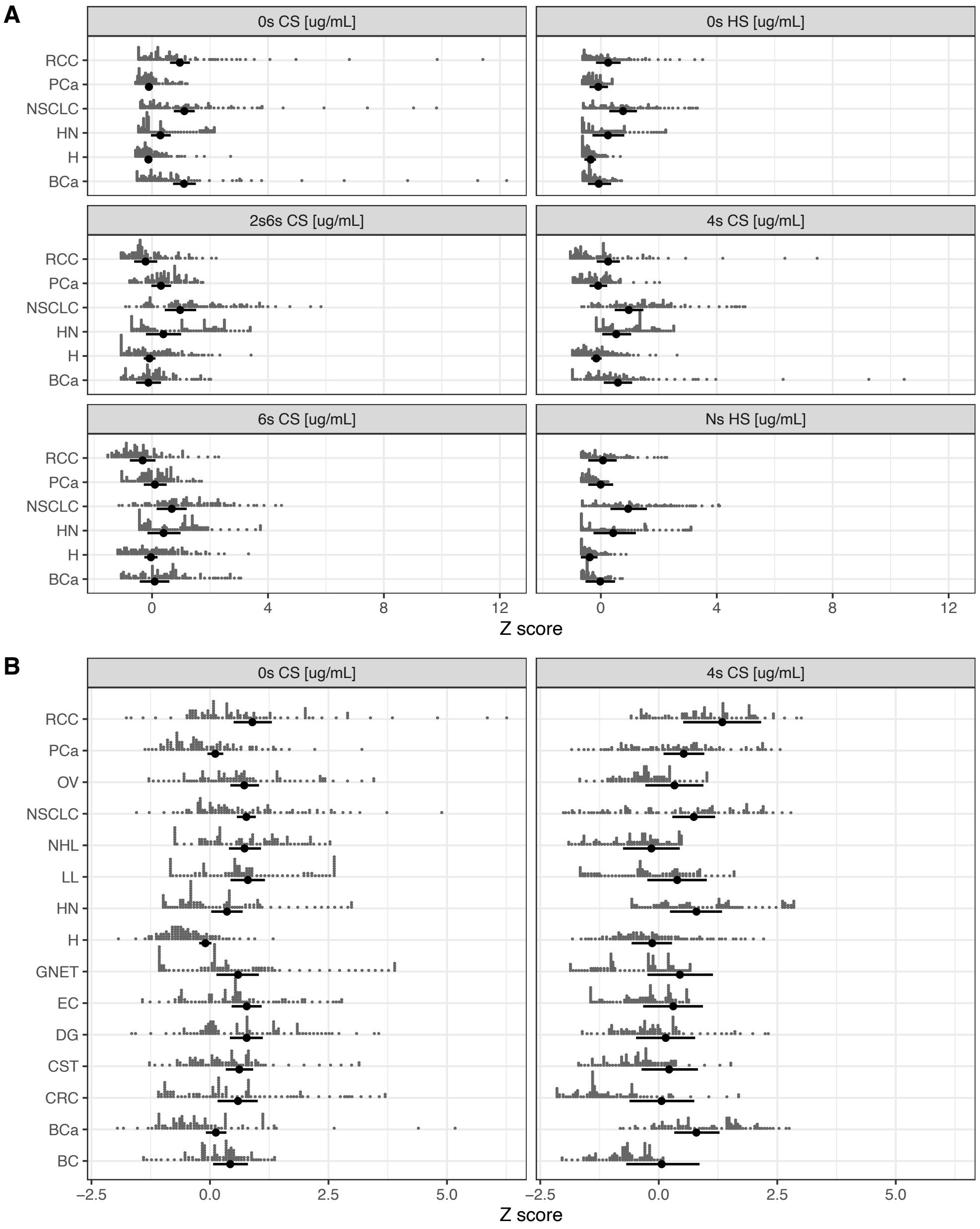

Fig. S1. Distributions of independently measured GAGome features (in µg mL-1) and their respective estimated posterior medians within individual cancer types and healthy subjects in the development study. Distributions of independently measured GAGome features (in µg mL^{-1}) and their respective estimated posterior medians within individual cancer types and healthy subjects in the development study. A) Urine free GAGomes (N = 560). B) Plasma free GAGomes (N = 942). Measured concentrations are shown as quantile dot plots in grey. Points in black show medians and lines show 95% credible interval. Key: CS - Chondroitin sulfate; HS – Heparan sulfate; H – healthy; BC – Breast cancer; BCa – Bladder cancer; CRC – Colorectal cancer; CST – Cervical cancer; DG - Diffuse Glioma; EC - Endometrial Carcinoma; GNET - Small Intestinal Neuroendocrine Tumor; HN - Head and Neck Cancer; LL - Chronic lymphocytic

leukemia; NHL - Diffuse large B-cell lymphoma; NSCLC - Non-small-cell Lung Carcinoma; OC - Ovarian Carcinoma; PCa – Prostate cancer; RCC - Renal Cell Carcinoma

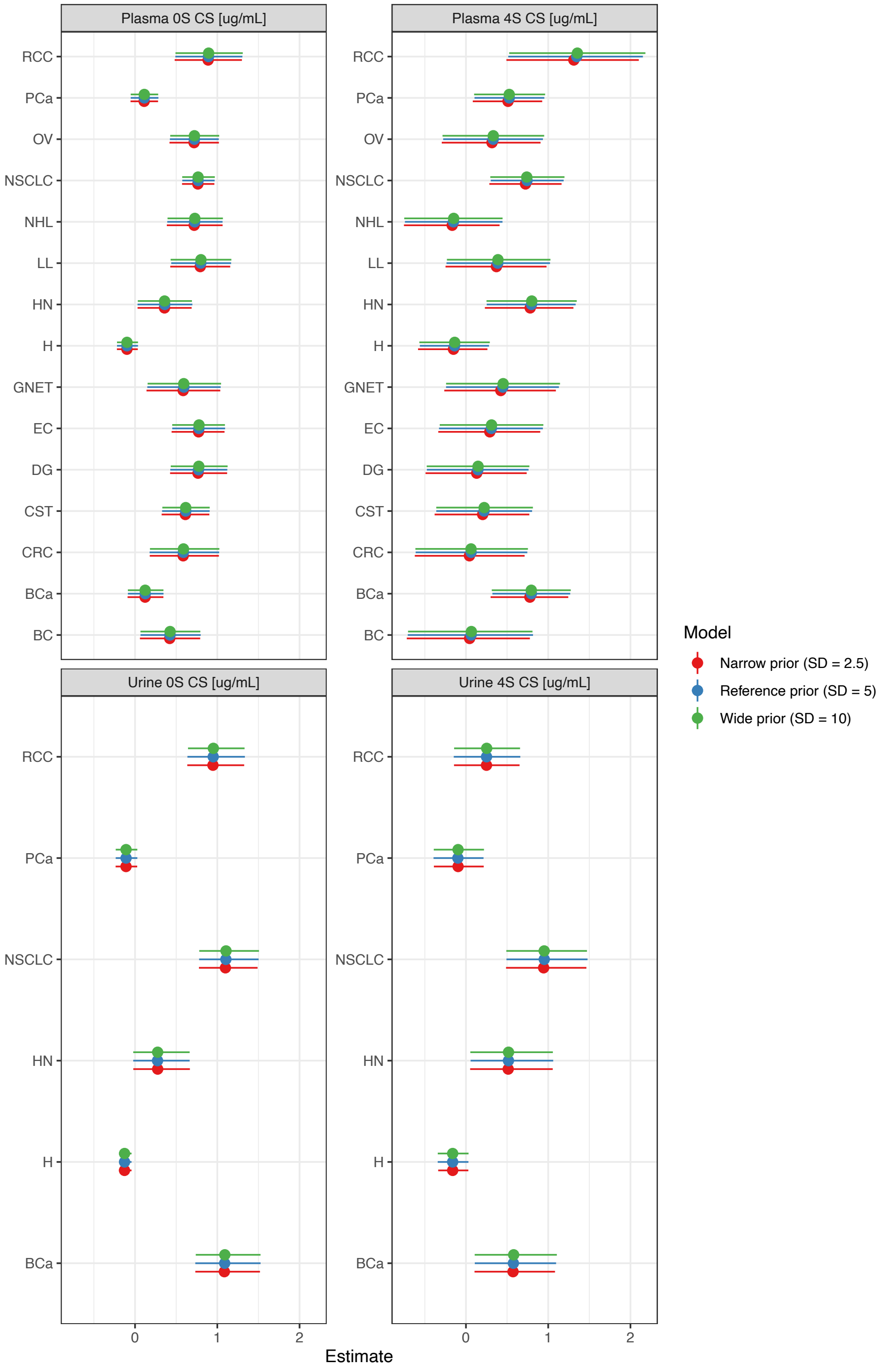

Fig. S2. Analysis of sensitivity of group estimates to the width of the prior distribution. The points correspond to estimated parameter medians and the lines show the 99% credibility interval. N_{urine}= 560, N_{plasma} = 942. Key: CS - Chondroitin sulfate; H – healthy; BC – Breast cancer; BCa – Bladder cancer; CRC – Colorectal cancer; CST – Cervical cancer; DG - Diffuse Glioma; EC - Endometrial Carcinoma; GNET - Small Intestinal Neuroendocrine Tumor; HN - Head and Neck Cancer; LL - Chronic lymphocytic leukemia; NHL - Diffuse large B-cell lymphoma; NSCLC - Non-small-cell Lung Carcinoma; OC - Ovarian Carcinoma; PCa – Prostate cancer; RCC - Renal Cell Carcinoma

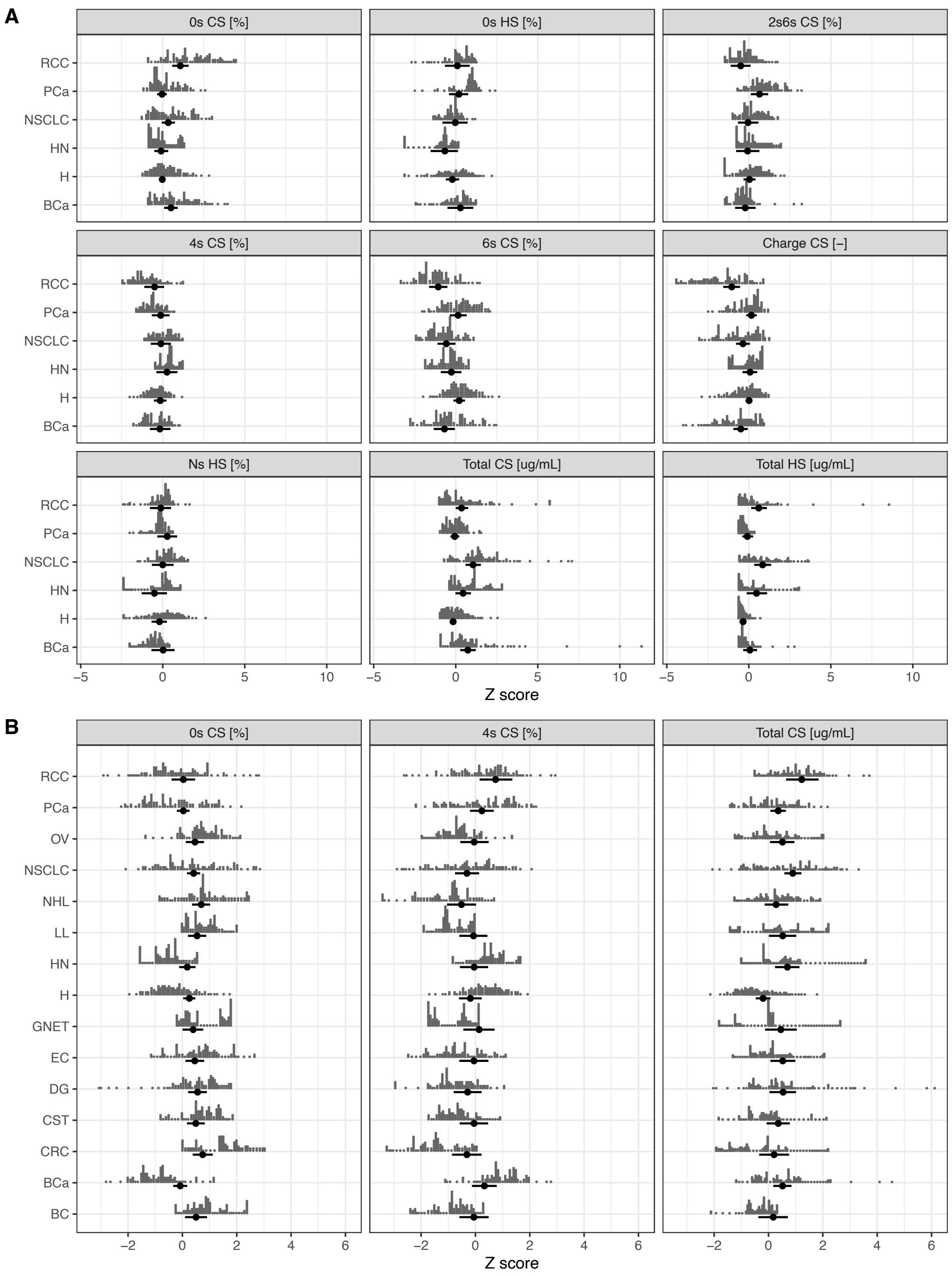

Fig. S3. Distributions of calculated GAGome features and their respective estimated posterior medians within individual cancer types and healthy subjects in the development study. A) Urine free GAGomes (N_{urine}= 560). B) Plasma free GAGomes (N_{plasma} = 942). Measured concentrations are shown as quantile dot plots in grey. Points in black show medians and lines show 95% credible interval. Key: CS - Chondroitin sulfate; HS – Heparan sulfate; H – healthy; BC – Breast cancer; BCa – Bladder cancer; CRC – Colorectal cancer; CST – Cervical cancer; DG - Diffuse Glioma; EC - Endometrial Carcinoma; GNET - Small Intestinal Neuroendocrine Tumor; HN - Head and Neck Cancer; LL - Chronic lymphocytic leukemia; NHL - Diffuse large B-cell

lymphoma; NSCLC - Non-small-cell Lung Carcinoma; OC - Ovarian Carcinoma; PCa – Prostate cancer; RCC - Renal Cell Carcinoma.

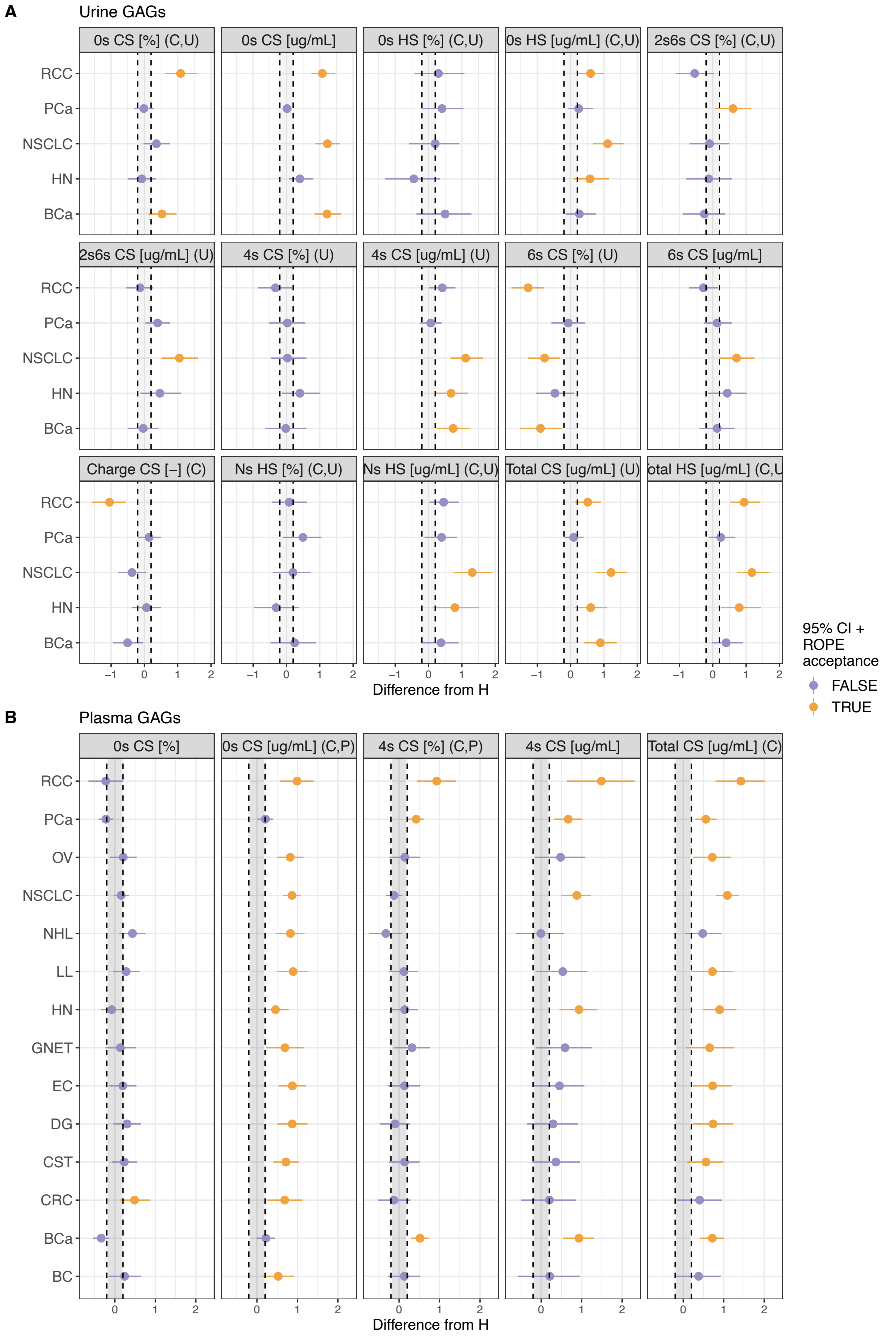

Fig. S4. Standardized differences in regression coefficients of GAGome features in each cancer type vs healthy subject group in the development study. A) Urine free GAGomes. B) Plasma free GAGomes. Forest plots show medians with 95% credible intervals for the difference vs healthy subject group. Region of practical equivalence (ROPE) is defined on the (-0.2, 0.2) interval (grey area) on the standardized scale. Deviation is accepted (orange) if less than 5% of the 95% credible interval falls within the ROPE interval and rejected otherwise (purple). Facet labels indicate if a GAGome feature was included as a predictor in the urine (U), plasma (P), or combined (C) pan-cancer GAG score..Key: CS - Chondroitin sulfate; HS – Heparan sulfate; H – healthy; BC – Breast cancer; BCa – Bladder cancer; CRC – Colorectal cancer; CST – Cervical cancer; DG - Diffuse Glioma; EC - Endometrial Carcinoma; GNET - Small Intestinal Neuroendocrine Tumor; HN - Head and Neck Cancer; LL - Chronic lymphocytic leukemia; NHL -

Diffuse large B-cell lymphoma; NSCLC - Non-small-cell Lung Carcinoma; OC - Ovarian Carcinoma; PCa – Prostate cancer; RCC - Renal Cell Carcinoma

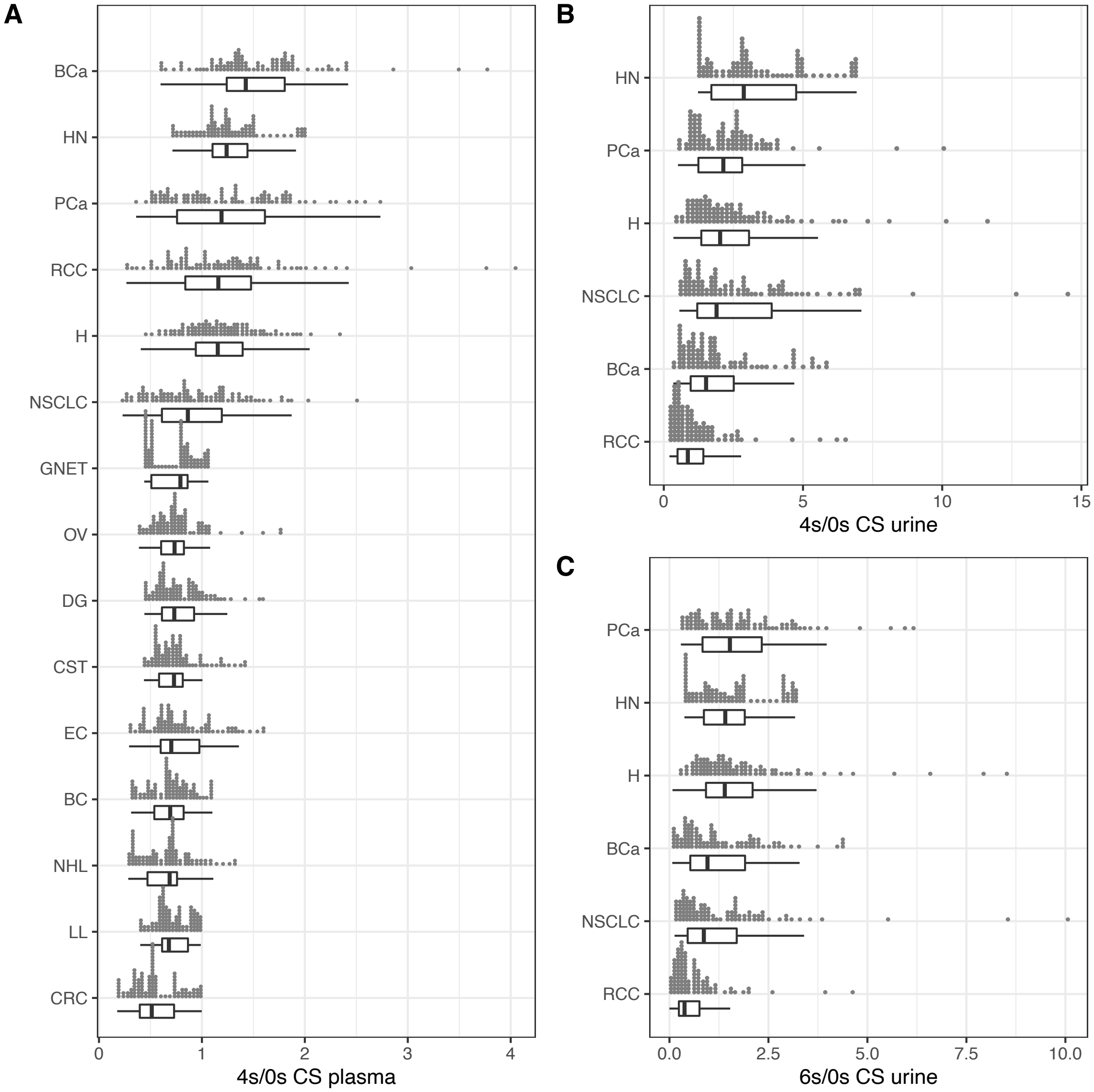

Fig. S5. Chondroitin sulfate (CS) ratios within individual cancer types and healthy subjects in the development study. A) Plasma 4S CS/0S CS ratio. B) Urine 4S CS/0S CS. C) 6S CS/0S CS ratios. Measured concentrations are shown as quantile dot plots in grey..Key: CS - Chondroitin sulfate; HS – Heparan sulfate; H – healthy; BC – Breast cancer; BCa – Bladder cancer; CRC – Colorectal cancer; CST – Cervical cancer; DG - Diffuse Glioma; EC - Endometrial Carcinoma; GNET - Small Intestinal Neuroendocrine Tumor; HN - Head and Neck Cancer; LL - Chronic lymphocytic leukemia; NHL - Diffuse large B-cell lymphoma; NSCLC - Non-small-cell Lung Carcinoma; OC - Ovarian Carcinoma; PCa – Prostate cancer; RCC - Renal Cell Carcinoma

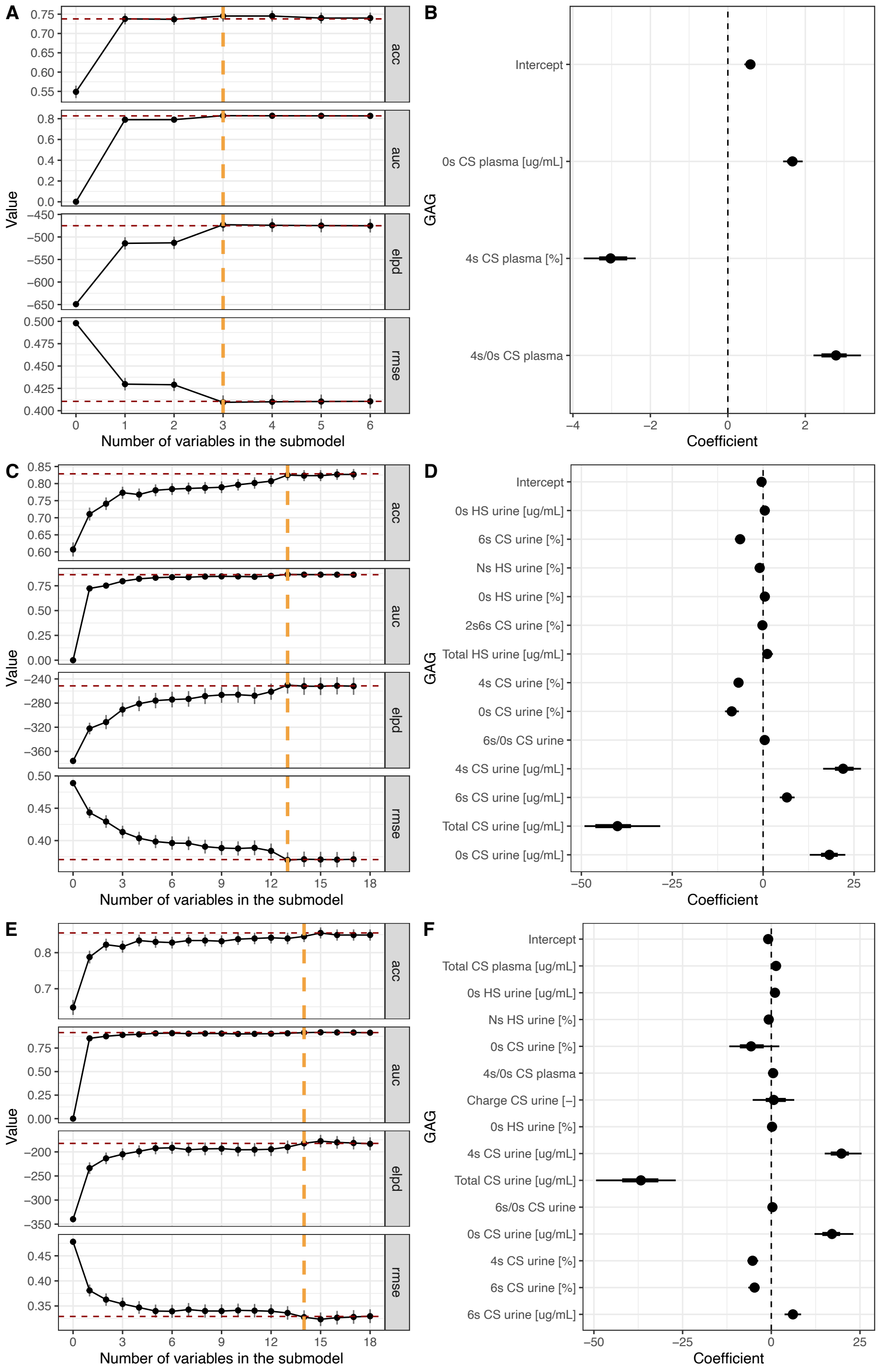

Fig. S6. Projection predictive score variable selection in the development study based on cross-validated accuracy metrics. A), C), E) Cross-validation variable selection for the plasma, urine, and combined free GAGome MCED score, respectively. Orange line shows the default suggested model size. B), D), F) Selected GAGome features and their coefficients. Points are medians of 95% credible interval, thick line shows 66% and thin line 95% credible interval. Key:

acc – accuracy; auc – area under the curve; elpd - expected log pointwise predictive density; rmse - root-mean-square error; CS – chondroitin sulfate; HS – heparan sulfate.

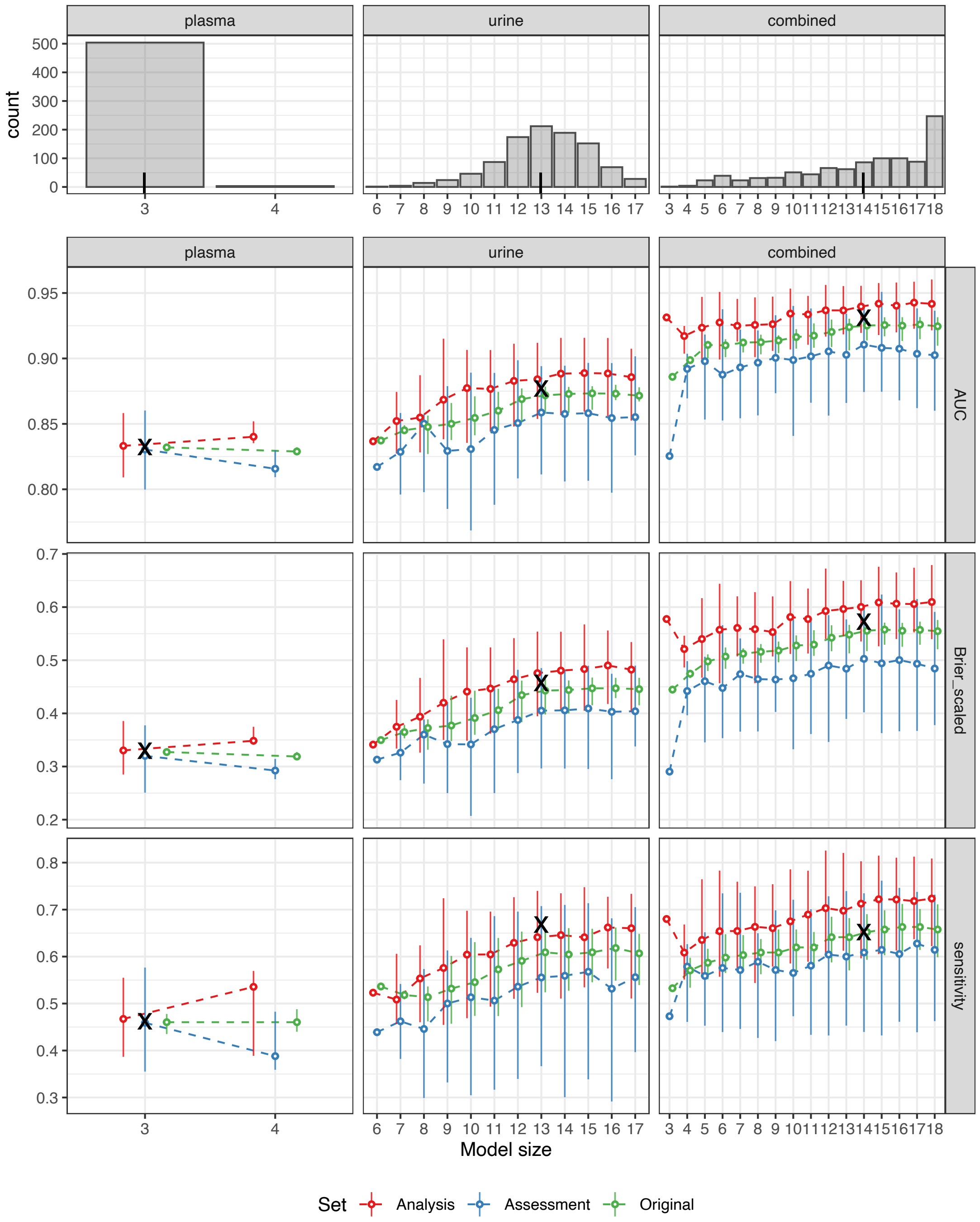

Fig. S7. Internal validation by bootstrapping of the plasma, urine, and combined free GAGome MCED score in the development study. We analyzed a total of 500 bootstraps for plasma, and 1000 for urine and combined datasets. In each bootstrap, we first fit the reference logistic Bayesian regression model and we carried out the projection predictive variable selection using leave-one-out cross-validation. Top panel - the selected model size. Bottom panels - AUC, scaled Brier metric and sensitivity at 95% specificity, and for the full and projected model on the analysis (bootstrapped), original and assessment dataset. Black cross indicates metrics for the final model (see the main text and Fig. S5). Key: AUC – area under the curve.

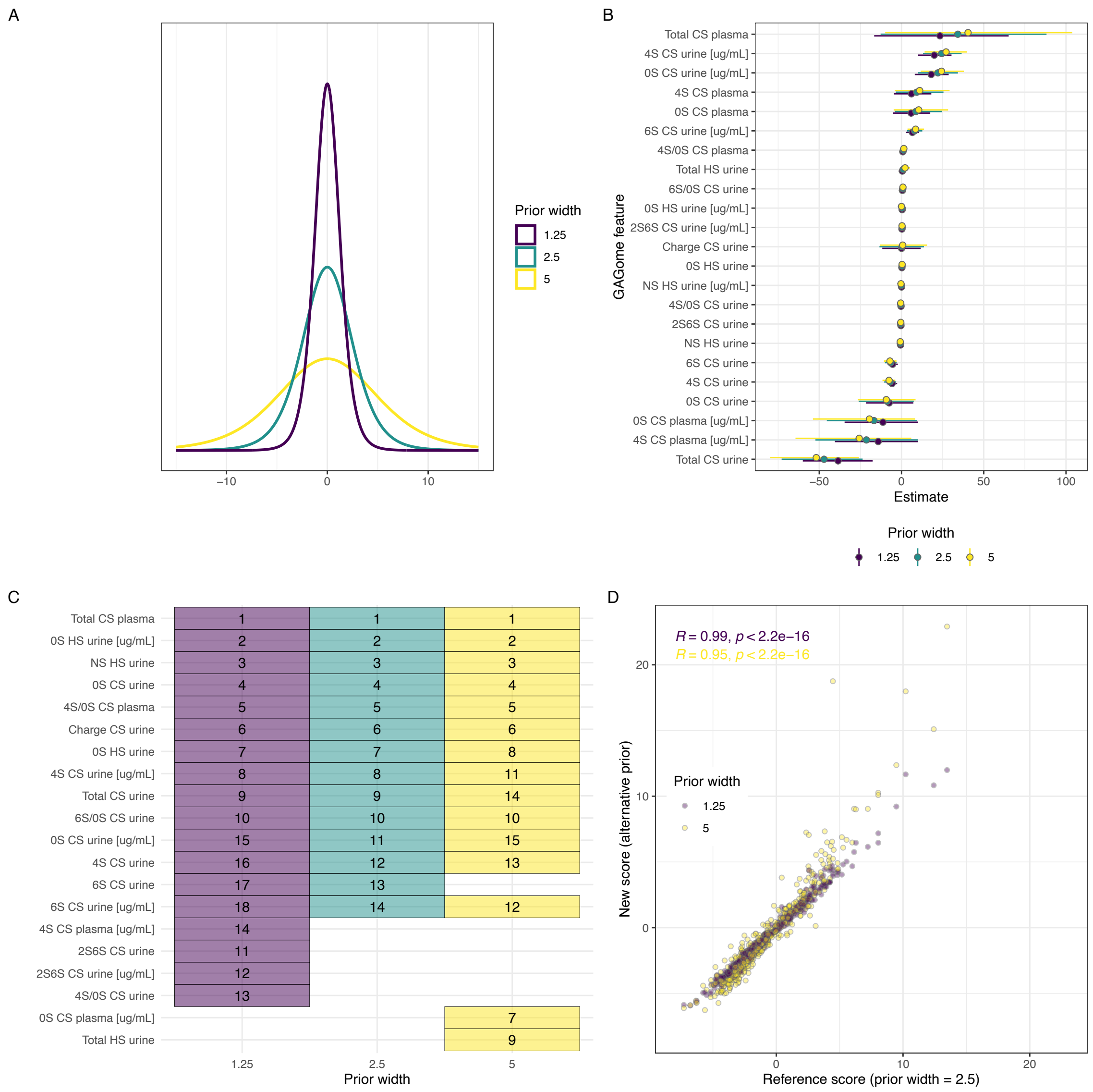

Fig. S8. Sensitivity analysis of the variable selection procedure using combined GAGome features. The model described in the main text is fit using prior width 2.5 (green in panels A-C). A) t-distribution priors with different priors (scale factors) used for to estimate standardized coefficient. B) Estimated coefficients in the reference model. C) Variable importance and model size resulting from alternative priors. Numbers in cells correspond to the variable importance in a respective model, after variable selection. D) Combined GAG score correlation between the reference score (y-axis, described in the main text) and the score derived from an alternative prior. R indicates the Pearson correlation coefficient between the two scores. Key: CS – chondroitin sulfate; HS – heparan sulfate.

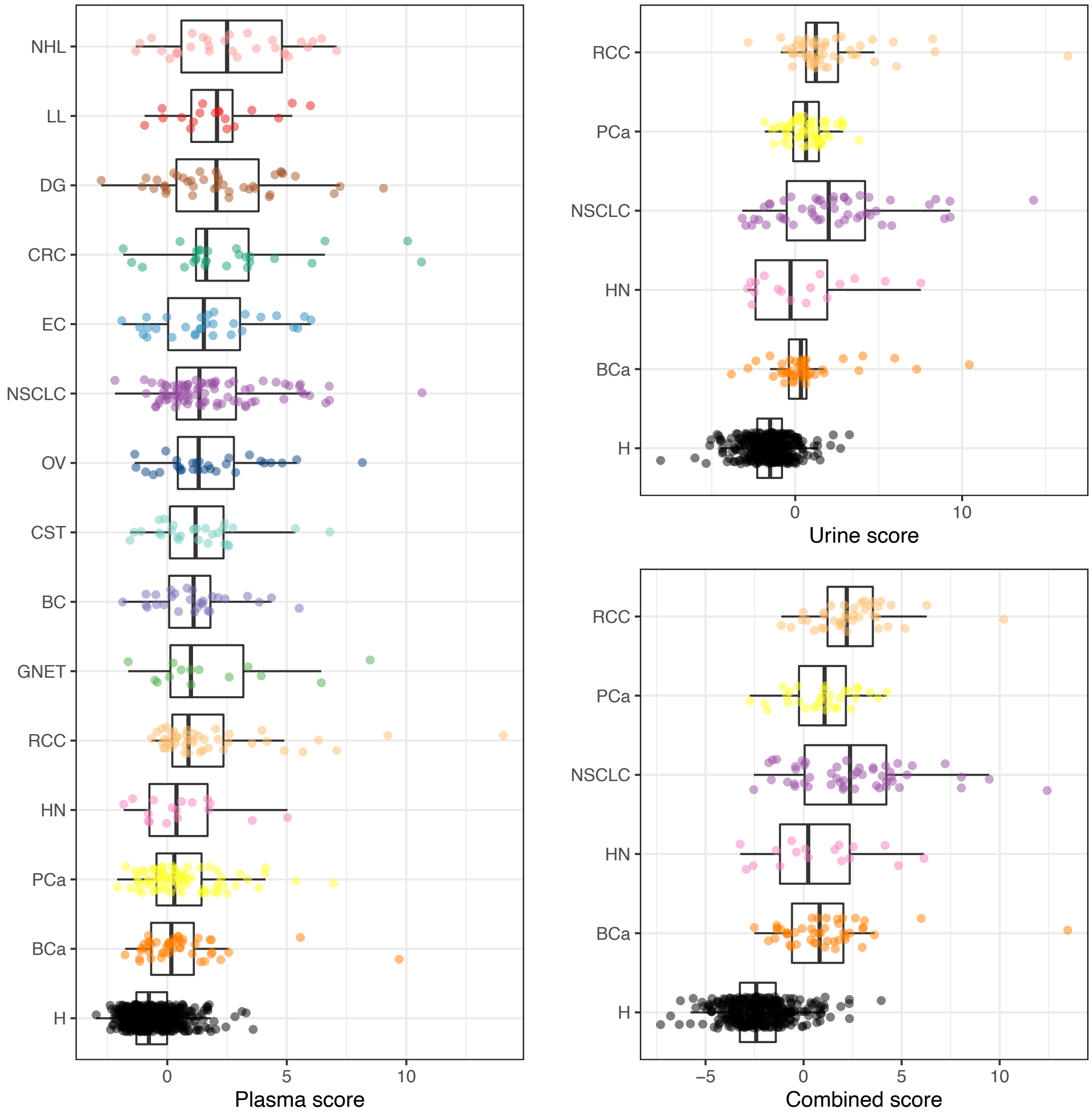

Fig. S9. Plasma, urine, and combined free GAGome MCED score across different cancer types in the development study. A) Plasma scores (N = 942). B) Urine scores (N = 560). C) Combined scores (N = 523). Key: H – healthy; BC – Breast cancer; BCa – Bladder cancer; CRC – Colorectal cancer; CST – Cervical cancer; DG - Diffuse Glioma; EC - Endometrial Carcinoma; GNET - Small Intestinal Neuroendocrine Tumor; HN - Head and Neck Cancer; LL - Chronic lymphocytic leukemia; NHL - Diffuse large B-cell lymphoma; NSCLC - Non-small-cell Lung Carcinoma; OC - Ovarian Carcinoma; PCa – Prostate cancer; RCC - Renal Cell Carcinoma.

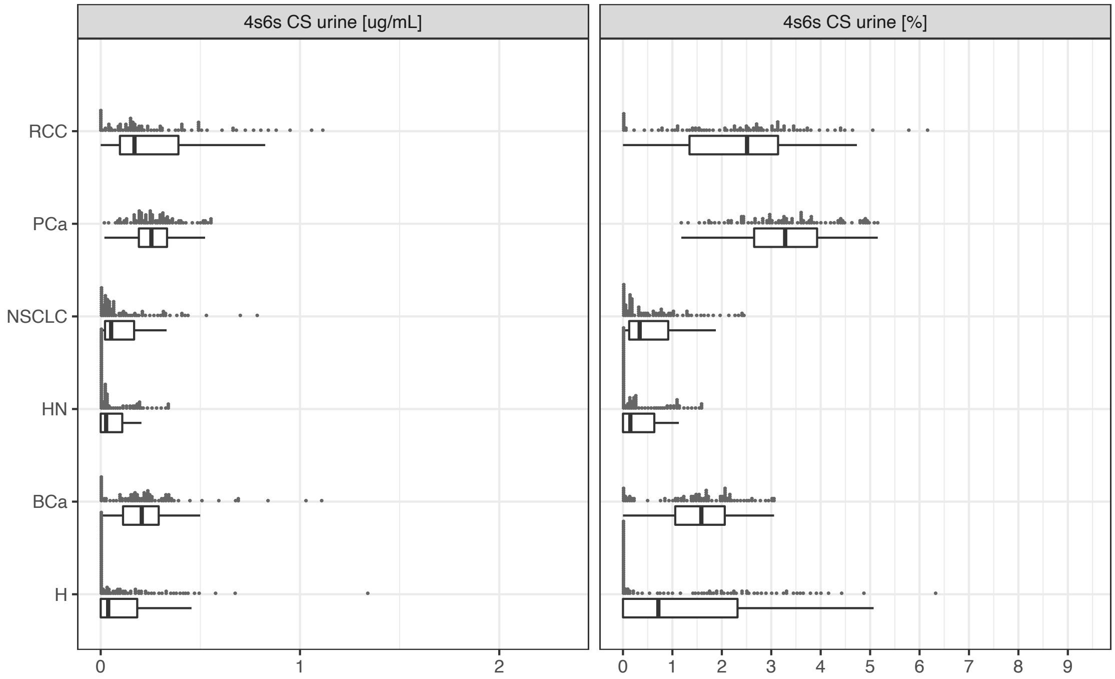

Fig. S10. Concentration (in µg mL-1) and fraction (%) of urine 4S6S CS in each cancer type and healthy subjects in the development study. Measured concentrations are shown as quantile dot plots in grey (N = 560). Key: H – healthy; BCa – Bladder cancer; HN - Head and Neck Cancer; NSCLC - Non-small-cell Lung Carcinoma; PCa – Prostate cancer; RCC - Renal Cell Carcinoma.

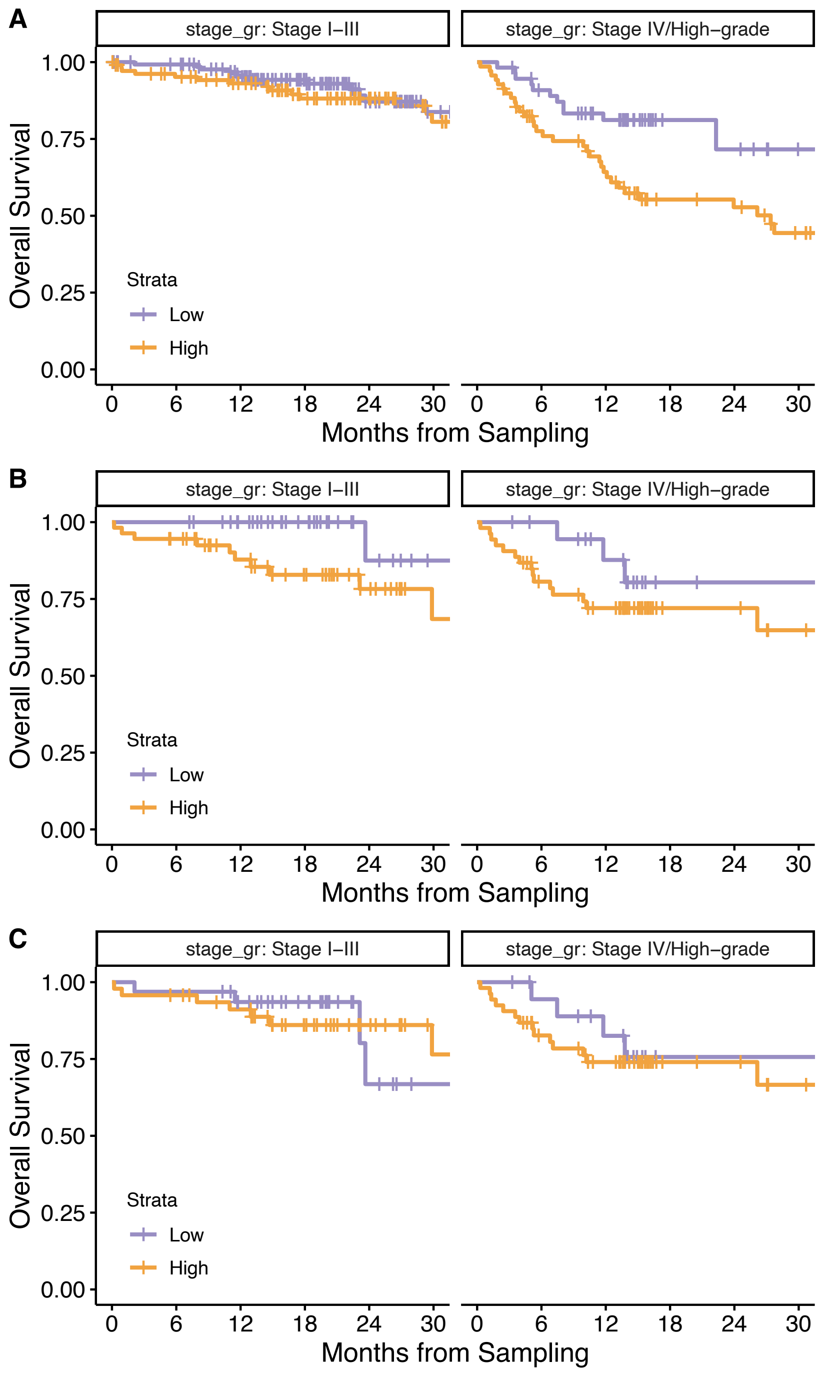

Fig. S11. Kaplan–Meier curves for overall survival across all cases, grouped by stage\grade, stratified into groups of "Low" and "High" based on free GAGome MCED scores using 95% specificity cut-off in the development study. For each score, the patients with scores higher than 98% specificity cut-off were assigned to the "High" (orange) vs. "Low" (purple) group. A) Plasma score (N = 370), B) urine score (N = 162), and C) combined score (N =152). Note: patients with unspecified stage/grade were grouped with Stage I-III.

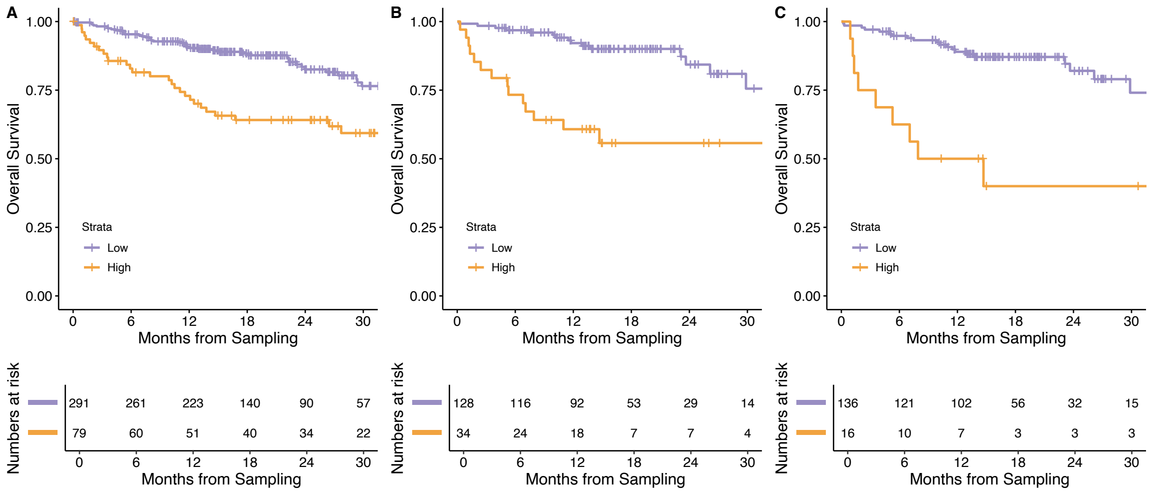

Fig. S12. Kaplan–Meier curves for overall survival across all cancer patients stratified into groups of "Low" and "High" free GAGome MCED score using maximally selected rank statistic in the development study. For each score, the patients with scores higher than an optimal cut-off were assigned to the "High" (orange), vs. "Low" (purple) group. The panels show number at risk for each group. A) Plasma score (cut-off score = 2.92, HR = 1.87 [95% CI = 1.37-2.57], $p < 0.001$). N = 370, 13 cancer types), B) Urine score (cut-off = 3.29, HR = 2.54 [95% CI = 1.46-3.95], $p < 0.001$), N = 162, 4 cancer types). C) Combined score (cut-off = 4.53, HR = 2.79 [95% CI = 1.61-4.88], $p < 0.001$), N =152, 4 cancer types).

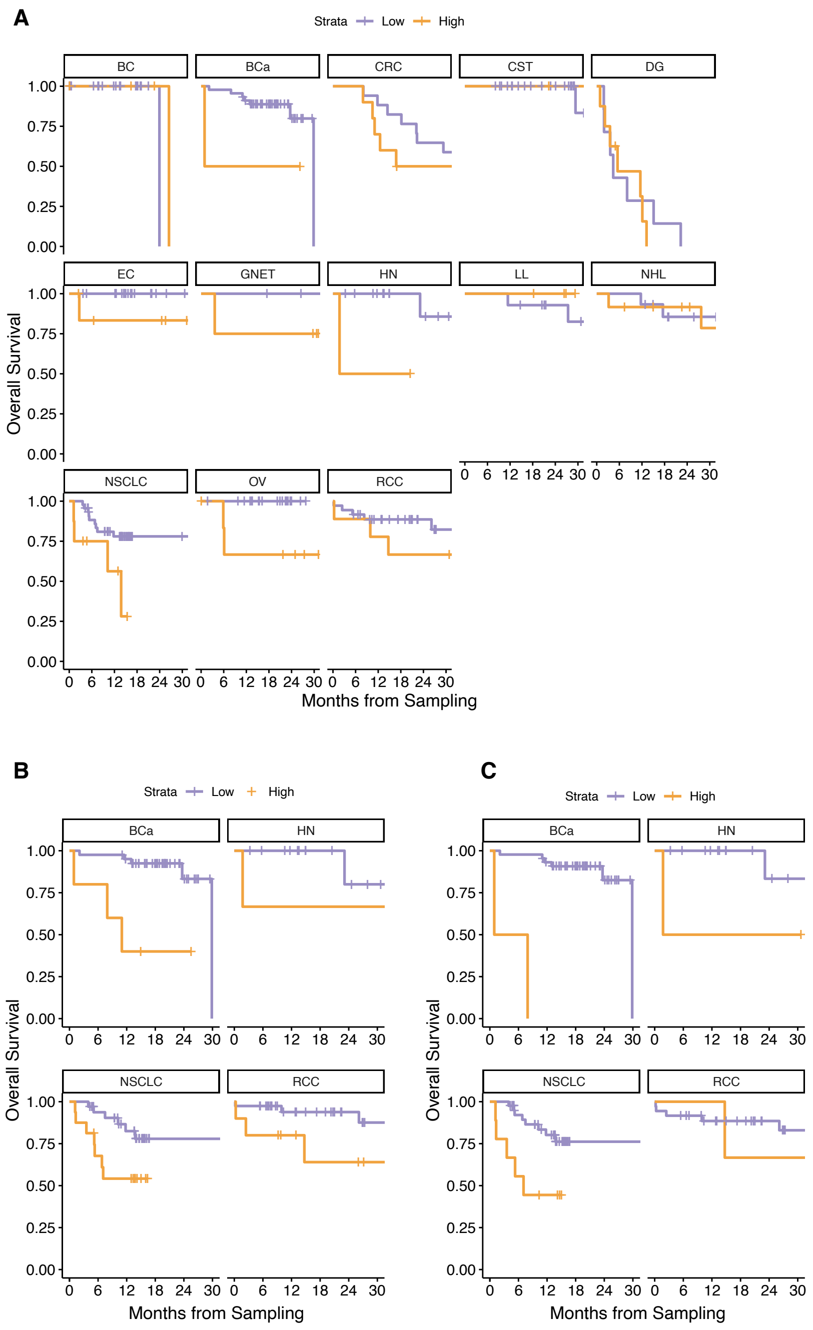

Fig. S13. Kaplan–Meier curves for overall survival across all cases within each cancer type, stratified into groups of "Low" and "High" based on free GAGome MCED scores using maximally selected rank statistic in the development study. For each score, the patients with scores higher than an optimal cut-off were assigned to the "High" (orange) vs. "Low" (purple) group. A) Plasma score (N = 370), B) urine score (N = 162), and C) combined score (N =152) above/below an optimal cut-off. Key: H – healthy; BC – Breast cancer; BCa – Bladder cancer; CRC – Colorectal cancer; CST – Cervical cancer; DG - Diffuse Glioma; EC - Endometrial

Carcinoma; GNET - Small Intestinal Neuroendocrine Tumor; HN - Head and Neck Cancer; LL - Chronic lymphocytic.

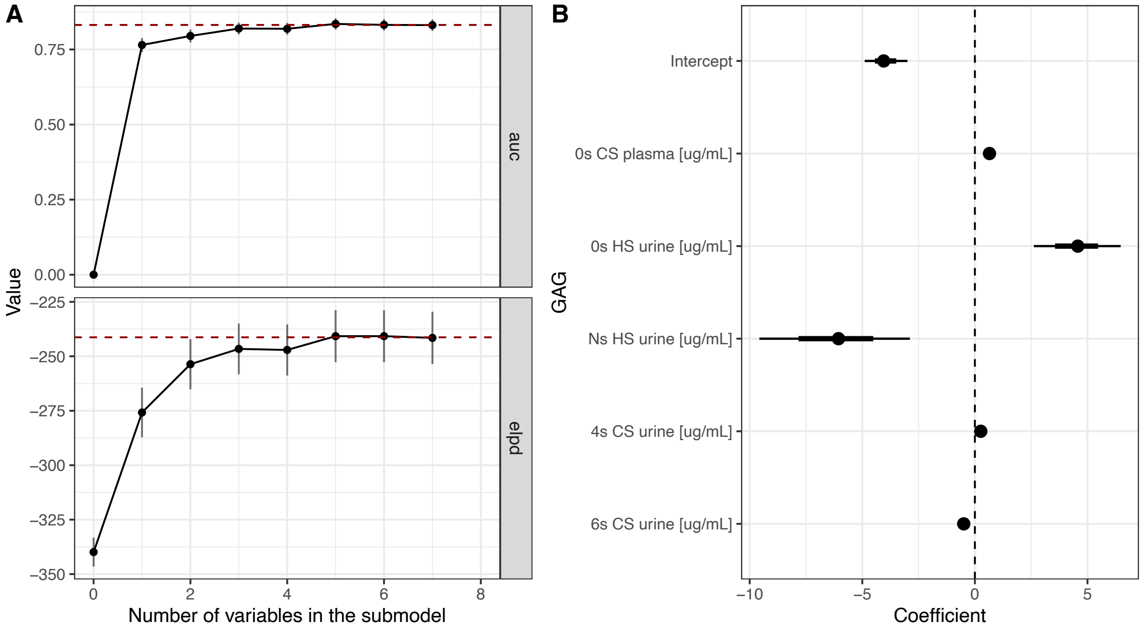

Fig. S14. Projection predictive score variable selection for the pruned combined free GAGome MCED score based on cross-validated accuracy metrics in the development study. A) Cross-validation variable selection for the pruned score. Orange line shows the default suggested model size. B) Selected GAGome features and their coefficients. Points are medians of 95% credible interval, thick line shows 66% and thin line 95% credible interval. Key: auc – area under the curve; elpd – expected log pointwise predictive density; CS – chondroitin sulfate; HS – heparan sulfate.

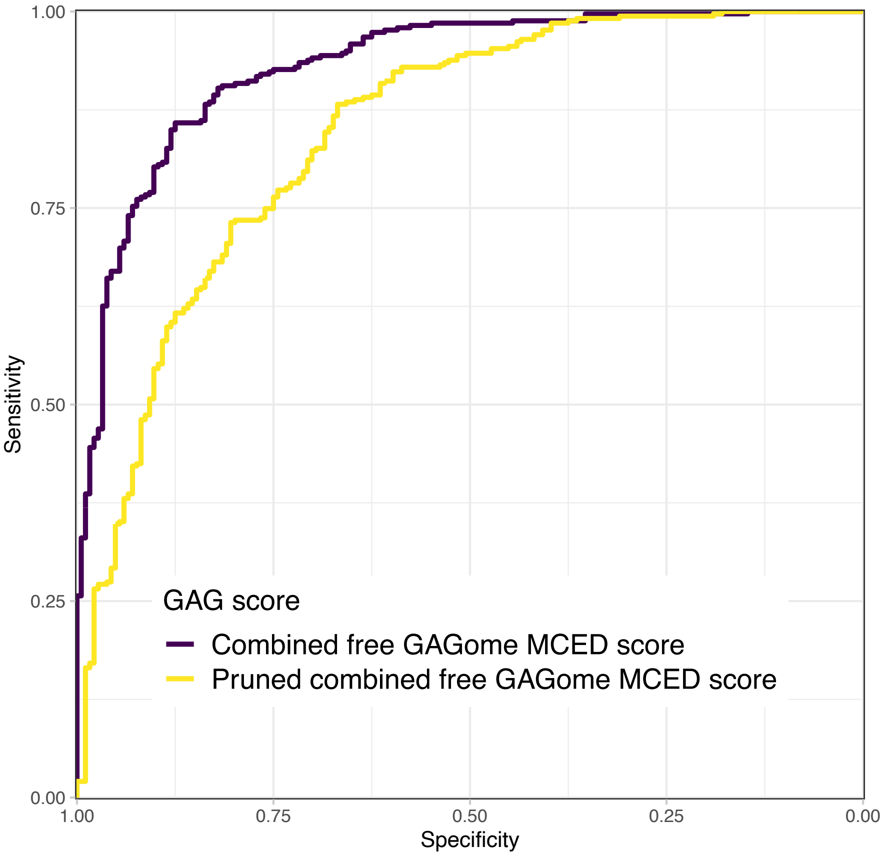

Fig. S15. Comparative performance of the combined and the pruned combined free GAGome MCED score in the development cohort for discriminating healthy controls vs any-cancer. The AUC values were AUC = 0.93 (95% CI = 0.9-0.95) and 0.85 (95% CI= 0.81-0.88) for the combined and the pruned combined free GAGome MCED score, respectively.

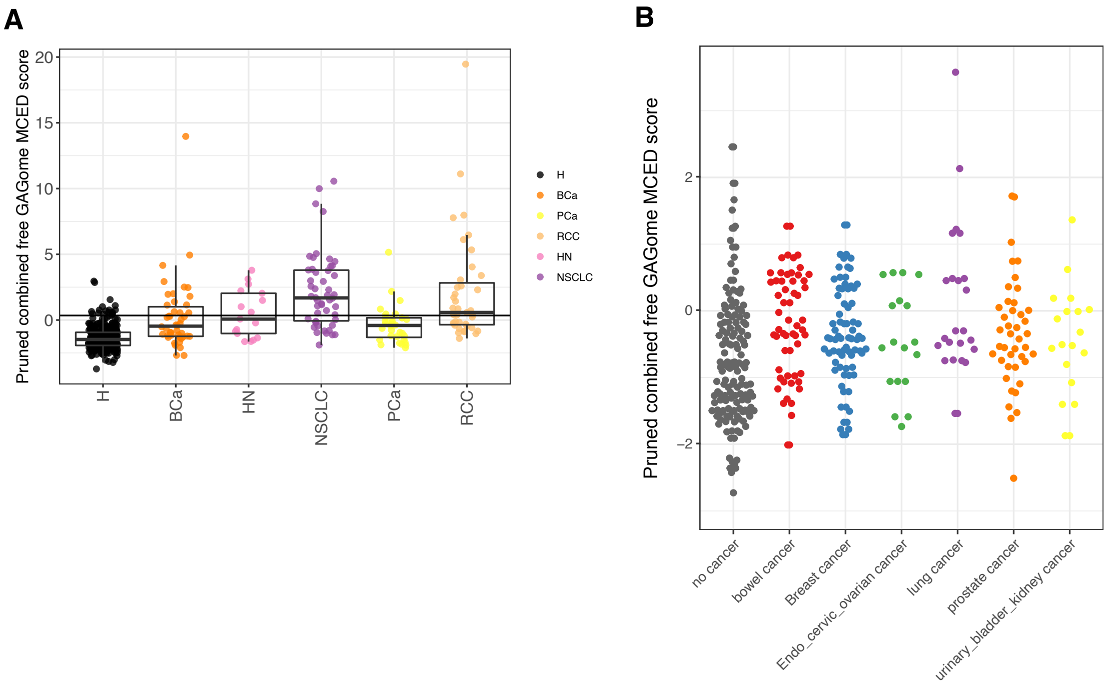

Fig. S16. Pruned combined free GAGome MCED score by cancer type. A) Score performance in the development study (N = 523). B) Score performance in the validation study (N = 281). Key: H – healthy; BCa – Bladder cancer; HN - Head and Neck Cancer; NSCLC - Non-small-cell Lung Carcinoma; PCa – Prostate cancer; RCC - Renal Cell Carcinoma.

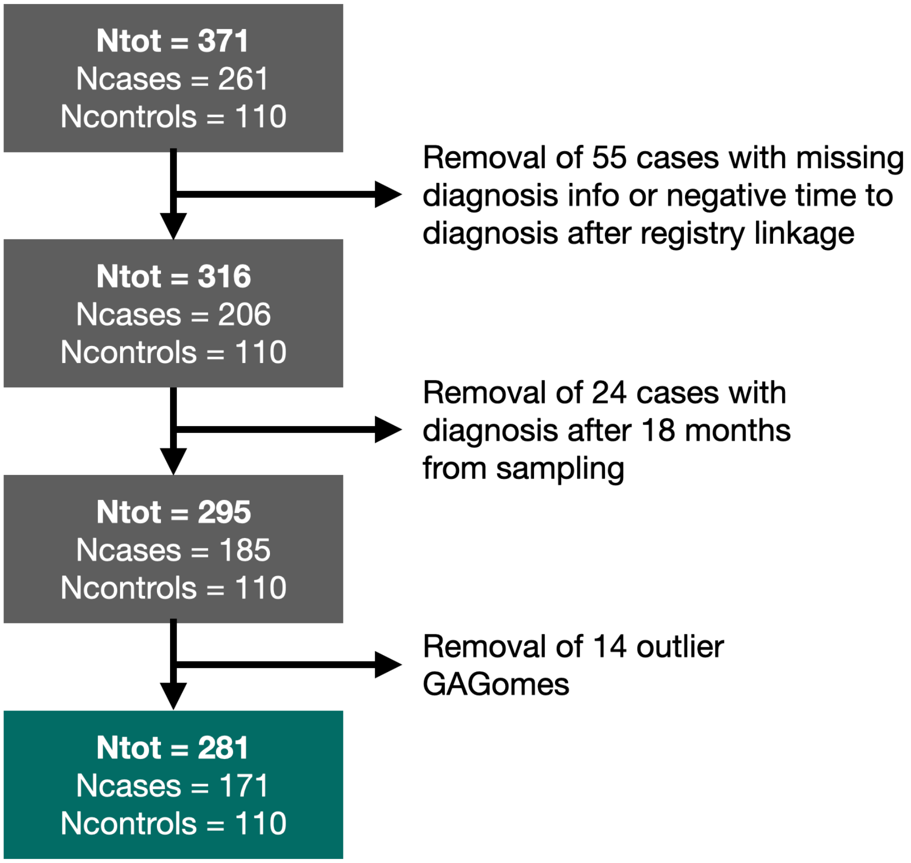

Fig. S17. Subject flow chart in the validation study.

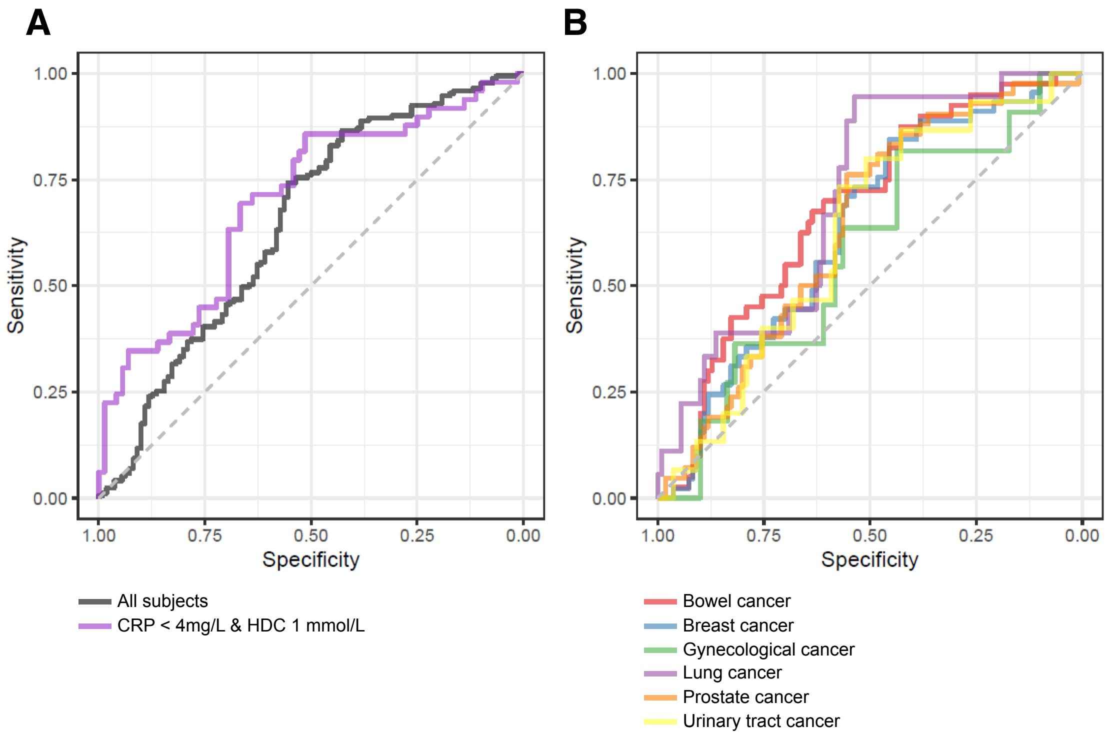

Fig. S18. Comparative performance of the pruned combined free GAGome MCED score in the overall population and relevant subsets in the validation study. ROC curves for discriminating cases (any-cancer in 18 months) vs controls (no cancer in 18 months) aggregating cases across all cancer types (left) or by cancer type (right).

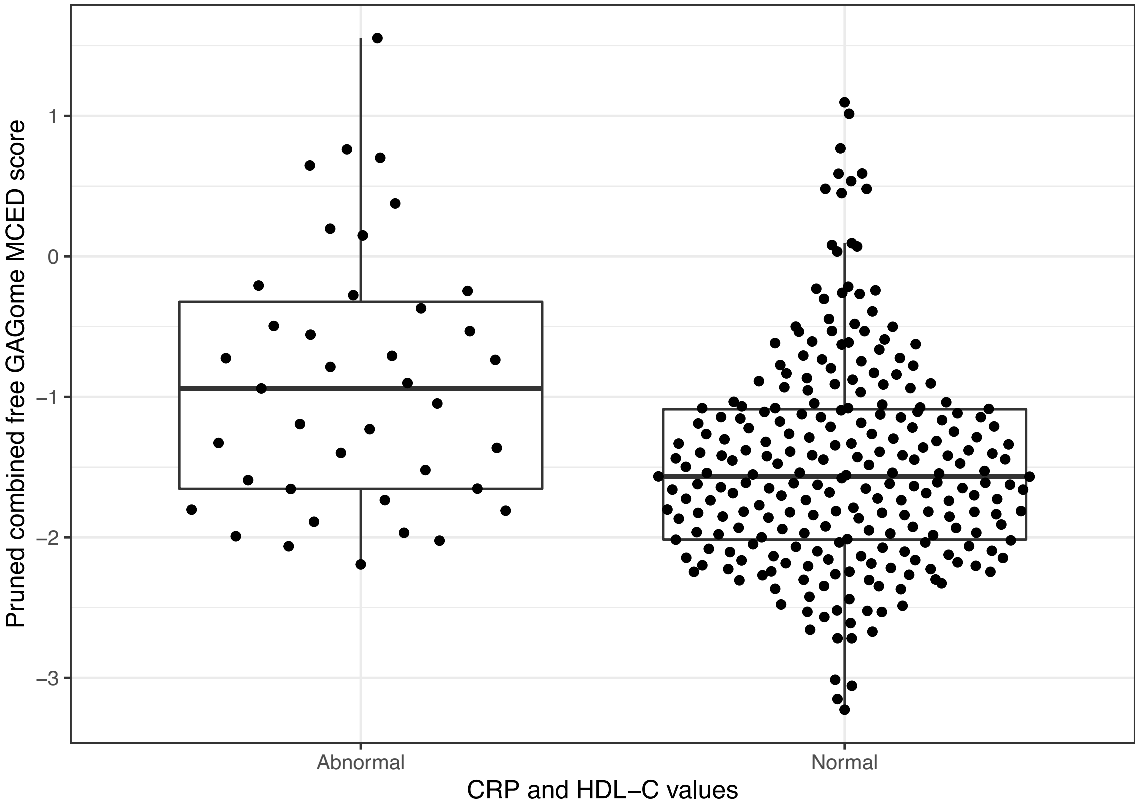

Fig. S19. The correlation between the pruned score and CRP or HDL-C in the healthy controls of the development study. Blood biomarker levels were considered abnormal if CRP was higher than 4 mg dL^{-1} or if HDL-C was below 1 mmol L^{-1} ($N_{abnormal}$ = 39, N_{normal} = 254). The mean GAG score in the abnormal group was -0.89 vs -1.48 (+60 %, t = 3.8236, p = 0.0004 , two-sided t-test).

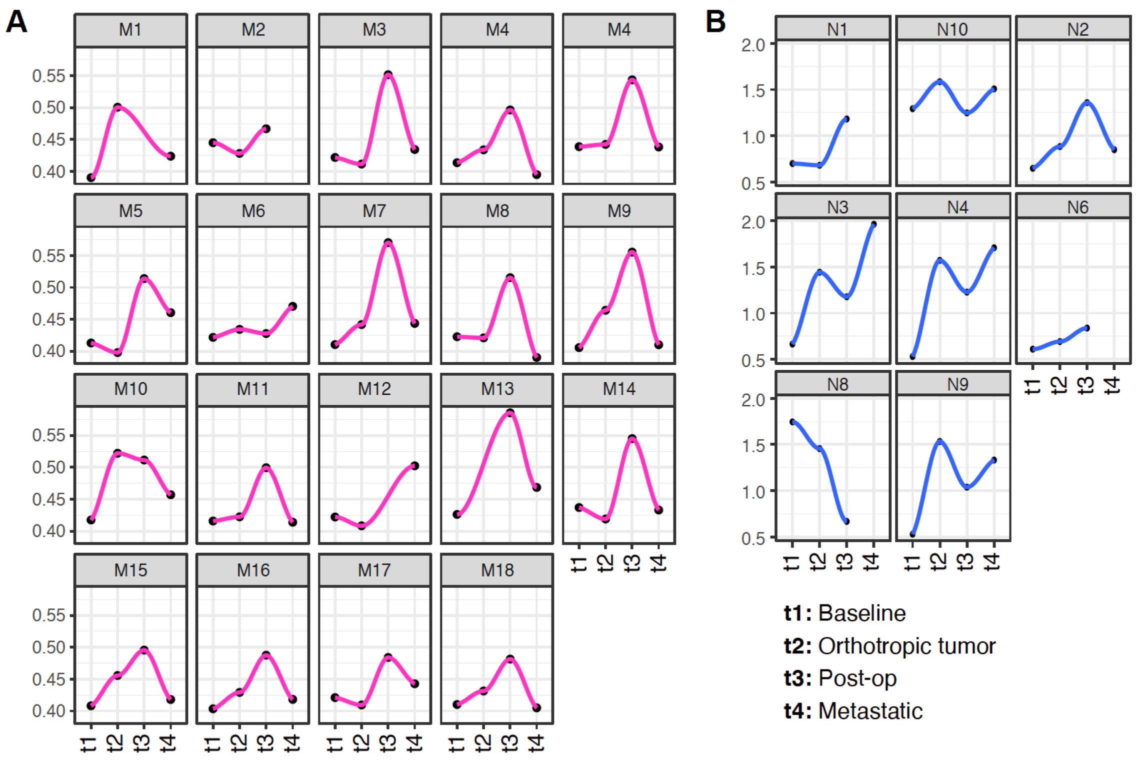

Fig. S20. Alterations in 4S CS (in µg mL-1) during cancer progression in the mice experiment. A) Plasma 4S CS in individual mice ("M") across time-points. B) Urine 4S CS changes in metabolic cages ("N") across time-points.

Table S1. Sample availability by stage/grade group in the development study

Stage/grade group	Combined (N=523)	Plasma (N=942)	Urine (N=560)
Healthy controls	339 (64.8%)	425 (45.1%)	340 (60.7%)
Stage I or Low grade	44 (8.4%)	178 (18.9%)	53 (9.5%)
Stage II	16 (3.1%)	54 (5.7%)	18 (3.2%)
Stage III	19 (3.6%)	57 (6.1%)	21 (3.8%)
Stage IV or High grade	105 (20.1%)	217 (23.0%)	126 (22.5%)
Unspecified stage/grade	0 (0%)	11 (1.2%)	2 (0.4%)

Table S2. Measured GAGome feature ranges in the development study. In bold, GAGome features deemed detectable (median concentration > 0.1 µg mL^{-1})

Fluid	GAGome feature	Median	Standard deviation	Minimum	Maximum
Plasma	**Total CS [µg mL^{-1}]**	9.100	2.950	2.079	23.253
	**4S CS [µg mL^{-1}]**	4.807	1.811	1.357	10.392
	**0S CS [µg mL^{-1}]**	4.067	1.602	0.674	16.422
	6S CS [µg mL^{-1}]	0.050	0.033	0.000	0.289
	Total HS [µg mL^{-1}]	0.002	0.020	0.000	0.205
	0S HS [µg mL^{-1}]	0.000	0.002	0.000	0.042
	NS HS [µg mL^{-1}]]	0.000	0.001	0.000	0.006
	NS2S HS [µg mL^{-1}]	0.000	0.016	0.000	0.163
	NS6S HS [µg mL^{-1}]	0.000	0.004	0.000	0.038
	TriS CS [µg mL^{-1}]	0.000	0.064	0.000	0.764
	TriS HS [µg mL^{-1}]	0.000	0.008	0.000	0.076
	Total HA [µg mL^{-1}]	0.000	0.002	0.000	0.014
	2S CS [µg mL^{-1}]	0.000	0.036	0.000	0.569
	2S HS [µg mL^{-1}]	0.000	0.000	0.000	0.002
	2S4S CS [µg mL^{-1}]	0.000	0.000	0.000	0.004
	2S6S CS [µg mL^{-1}]	0.000	0.002	0.000	0.015
	2S6S HS [µg mL^{-1}]	0.000	0.001	0.000	0.009
	4S6S CS [µg mL^{-1}]	0.000	0.002	0.000	0.028
	6S HS [µg mL^{-1}]	0.000	0.000	0.000	0.004
Urine	**Total CS [µg mL^{-1}]**	8.866	8.116	1.164	90.043
	**4S CS [µg mL^{-1}]**	3.837	3.755	0.274	41.214
	**6S CS [µg mL^{-1}]**	2.399	1.446	0.000	8.549
	**0S CS [µg mL^{-1}]**	1.780	3.951	0.155	40.677
	**Total HS [µg mL^{-1}]**	0.504	1.357	0.000	16.913
	**0S HS [µg mL^{-1}]**	0.265	0.610	0.000	4.261
	**2S6S CS [µg mL^{-1}]**	0.158	0.144	0.000	0.946
	**NS HS [µg mL^{-1}]**	0.125	0.310	0.000	2.092
	4S6S CS [µg mL^{-1}]	0.076	0.204	0.000	2.332
	2S6S HS [µg mL^{-1}]	0.031	0.168	0.000	1.473
	2S4S CS [µg mL^{-1}]	0.026	0.054	0.000	0.481
	Total HA [µg mL^{-1}]	0.009	0.057	0.000	0.443
	TriS CS [µg mL^{-1}]	0.006	0.031	0.000	0.290
	6S HS [µg mL^{-1}]	0.006	0.035	0.000	0.235
	2S CS [µg mL^{-1}]	0.001	0.042	0.000	0.676
	2S HS [µg mL^{-1}]	0.000	0.001	0.000	0.003
	NS2S HS [µg mL^{-1}]	0.000	0.058	0.000	0.720
	NS6S HS [µg mL^{-1}]	0.000	0.038	0.000	0.437
	TriS HS [µg mL^{-1}]	0.000	0.644	0.000	13.681

Table S3. Sensitivity at 95% specificity for each free GAGome MCED score within individual tumor stage or grades in the development cohort. Key: Key: H, healthy; S1/LG, stage I or low grade; S2, stage II; S3, stage III; S4/ HG, stage IV or high grade.

Score	Stage	AUC [95% CI]	Sensitivity at 95% specificity [95% CI]	Sensitivity at 99% specificity [95% CI]
Plasma	S1/LG	0.81 [0.77-0.84]	0.416 [0.342-0.492]	0.219 [0.161-0.287]
Plasma	S1/LG-S2	0.81 [0.78-0.85]	0.422 [0.358-0.489]	0.22 [0.168-0.279]
Plasma	S1/LG-S3	0.83 [0.8-0.86]	0.45 [0.392-0.509]	0.249 [0.2-0.303]
Plasma	Any stage	0.83 [0.81-0.86]	0.462 [0.419-0.506]	0.257 [0.22-0.297]
Urine	S1/LG	0.88 [0.83-0.94]	0.623 [0.479-0.752]	0.113 [0.043-0.23]
Urine	S1/LG-S2	0.88 [0.83-0.93]	0.634 [0.511-0.745]	0.141 [0.07-0.244]
Urine	S1/LG-S3	0.88 [0.84-0.93]	0.63 [0.523-0.729]	0.152 [0.086-0.242]
Urine	Any stage	0.88 [0.84-0.91]	0.668 [0.602-0.73]	0.25 [0.194-0.313]
Combined	S1/LG	0.93 [0.9-0.96]	0.614 [0.455-0.756]	0.136 [0.052-0.274]
Combined	S1/LG-S2	0.93 [0.9-0.96]	0.6 [0.465-0.724]	0.217 [0.121-0.342]
Combined	S1/LG-S3	0.93 [0.91-0.96]	0.595 [0.479-0.704]	0.253 [0.162-0.364]
Combined	Any stage	0.93 [0.91-0.95]	0.658 [0.584-0.726]	0.353 [0.284-0.427]

Table S2. Sensitivity at 95% and 99% specificity for each free GAGome MCED score within individual cancer types in the development cohort. Key: H – healthy; BC – Breast cancer; BCa – Bladder cancer; CRC – Colorectal cancer; CST – Cervical cancer; DG - Diffuse Glioma; EC - Endometrial Carcinoma; GNET - Small Intestinal Neuroendocrine Tumor; HN - Head and Neck Cancer; LL - Chronic lymphocytic leukemia; NHL - Diffuse large B-cell lymphoma; NSCLC - Non-small-cell Lung Carcinoma; OC - Ovarian Carcinoma; PCa – Prostate cancer; RCC - Renal Cell Carcinoma.

Score	Cancer type	Sensitivity at 95% specificity [95% CI]	Sensitivity at 99% specificity [95% CI]
Plasma	PCa	27.5 [16.2-37.5]	13.8 [3.8-27.5]
Plasma	HN	29.4 [11.8-52.9]	11.8 [0-29.4]
Plasma	NSCLC	50.6 [36.1-62.7]	30.1 [16.9-45.8]
Plasma	BCa	23.4 [10.6-38.3]	6.4 [0-21.3]
Plasma	RCC	42.2 [26.7-60]	24.4 [11.1-40.1]
Plasma	DG	60 [45-77.5]	42.5 [22.5-67.5]
Plasma	CRC	70.4 [44.4-92.6]	40.7 [22.2-63]
Plasma	NHL	66.7 [50-83.3]	50 [30-70]
Plasma	CST	50 [28.6-71.4]	17.9 [0-50]
Plasma	EC	60 [36.7-80]	33.3 [13.3-53.3]
Plasma	GNET	42.9 [14.3-71.4]	28.6 [7.1-57.1]
Plasma	OV	53.3 [33.3-73.3]	33.3 [13.3-53.3]
Plasma	LL	66.7 [44.4-88.9]	38.9 [5.6-72.2]
Plasma	BC	46.4 [25-67.9]	17.9 [3.6-39.3]
Urine	PCa	64.3 [42.9-80.4]	12.5 [1.8-41.1]
Urine	HN	47.1 [23.5-70.6]	23.5 [5.9-52.9]
Urine	NSCLC	70 [56-82]	52 [32-70]
Urine	BCa	52.2 [26.1-69.6]	15.2 [4.3-28.3]
Urine	RCC	82.4 [64.7-92.2]	33.3 [15.7-60.8]
Combined	PCa	62.5 [43.8-81.2]	25 [6.2-59.4]
Combined	HN	47.1 [23.5-70.6]	35.3 [11.8-64.7]
Combined	NSCLC	70 [56-82]	56 [36-76]
Combined	BCa	56.5 [39.1-76.1]	23.9 [8.7-52.2]
Combined	RCC	84.6 [71.8-97.4]	56.4 [33.3-84.6]

Table S5. Number of samples within and outside the visualized limits in Figure 1 for each free GAGome MCED score. Cancers with unspecified stage/grade were omitted from Figure 1.

Stage/grade group	Combined		Plasma		Urine	
	Score inside (-6,6) (N=510)	Score outside (-6,6) (N=13)	Score inside (-6,6) (N=917)	Score outside (-6,6) (N=25)	Score inside (-6,6) (N=545)	Score outside (-6,6) (N=15)
Healthy controls	335	4	425	0	338	2
Stage I/Low-grade	44	0	175	3	51	2
Stage II	16	0	51	3	17	1
Stage III	16	3	50	7	18	3
Stage IV/High-grade	99	6	205	12	119	7
Unspecified	0	0	11	0	2	0

Table S6. Pruned combined free GAGome MCED score performance metrics in the validation study.

	Controls	Cases	AUC [95% CI]	Sensitivity at 95% specificity [95% CI]	Sensitivity at 99% specificity [95% CI]
**Pruned combined free GAGome MCED score (overall population)**					
All cases	110	171	0.65 [0.58-0.72]	-	-
Stage 0-II	110	107	0.62 [0.54-0.69]	-	-
Stage III-IV	110	51	0.73 [0.65-0.82]	-	-
Diagnosis < 3 mo.	110	35	0.69 [0.61-0.78]	-	-
Diagnosis < 6 mo.	110	68	0.67 [0.59-0.75]	-	-
Diagnosis < 12 mo.	110	140	0.64 [0.58-0.72]	-	-
Bowel cancer	110	40	0.68 [0.59-0.77]	-	-
Urinary tract cancer	110	15	0.62 [0.49-0.76]	-	-
Breast cancer	110	45	0.63 [0.54-0.73]	-	-
Prostate cancer	110	42	0.64 [0.55-0.73]	-	-
Lung cancer	110	18	0.71 [0.59-0.82]	-	-
Gynecological cancer	110	11	0.58 [0.40-0.75]	-	-
**Pruned combined free GAGome MCED score (subset population, CRP < 4 mg dL^{-1} and HDL-C > 1 mmol L^{-1})**					
All cases	72	49	0.70 [0.60-0.80]	30.1% [14.3%-46.9%]	22.6% [2%-38.8%]
Stage 0-II	72	34	0.71 [0.61-0.82]	32.3% [14.7%-47.1%]	23.53% [0-38.2%]
Stage I	72	19	0.73 [0.61-0.85]	26.3% [5.3%-47.4%]	21.05 [0%-42.1%]
Poor prognosis (death in <7.8 years from diagnosis)	72	7	0.88 [0.77-0.99]	42.9% [14.3%-85.7%]	42.9% [0%-71.4%]

Table S7. Statistically significant correlations between blood biochemistry variables and the pruned combined free GAGome MCED score in the validation study. Correlations were considered statistically significant if the p-value was below 0.05 after adjusting for multiple testing using the Holm method.

**Variable**	**Kendall correlation coefficient**	**p-value**	**Adjusted p-value**
hsCRP in heparin (mg/L)	0.293	$1.14*10^{-8}$	$2.3*10^{-6}$
Glucose in NaF (mmol/L)	0.198	$2.00*10^{-6}$	$3.91*10^{-4}$
Uric acid in heparin (mmol/L)	0.191	$2.24*10^{-4}$	$4.21*10^{-2}$
Triglycerides in heparin (mmol/L)	0.181	$8.14*10^{-6}$	$1.57*10^{-3}$
HDL Cholesterol in heparin (mmol/L)	-0.163	$1.07*10^{-4}$	$2.02*10^{-2}$

Dataset S1 (separate file). Detailed patients' characteristics in the development study.

Dataset S2 (separate file). Summary and diagnostics of Bayesian estimation models in the development study.

Dataset S3 (separate file). Summary and diagnostics of the reference Bayesian logistic models for the pan-cancer GAG plasma, urine, and combined scores in the development study.

SI References

1. Shao, C. et al. Comparative glycomics of leukocyte glycosaminoglycans. FEBS J 280, 2447–2461 (2013).
2. Schmidt, E. P. et al. Urinary Glycosaminoglycans Predict Outcomes in Septic Shock and Acute Respiratory Distress Syndrome. Am J Respir Crit Care Med 194, 439–449 (2016).
3. Schmidt, E. P. et al. The circulating glycosaminoglycan signature of respiratory failure in critically ill adults. J Biol Chem 289, 8194–8202 (2014).
